# Glucose oxidase‐instructed biomineralization of calcium‐based biomaterials for biomedical applications

**DOI:** 10.1002/EXP.20210110

**Published:** 2023-07-11

**Authors:** Lian‐Hua Fu, Chao Qi, Tuanwei Sun, Kai Huang, Jing Lin, Peng Huang

**Affiliations:** ^1^ Marshall Laboratory of Biomedical Engineering International Cancer Center, Laboratory of Evolutionary Theranostics (LET) School of Biomedical Engineering Shenzhen University Medical School Shenzhen University Shenzhen China; ^2^ Department of Materials Science and Engineering University of Toronto Toronto Ontario Canada

**Keywords:** biomimetic mineralization, calcium‐based biomaterials, cancer therapy, diabetes, glucose oxidase

## Abstract

In recent years, glucose oxidase (GOx) has aroused great research interest in the treatment of diseases related to abnormal glucose metabolisms like cancer and diabetes. However, as a kind of endogenous oxido‐reductase, GOx suffers from poor stability and system toxicity in vivo. In order to overcome this bottleneck, GOx is encapsulated in calcium‐based biomaterials (CaXs) such as calcium phosphate (CaP) and calcium carbonate (CaCO_3_) by using it as a biotemplate to simulate the natural biomineralization process. The biomineralized GOx holds improved stability and reduced side effects, due to the excellent bioactivity, biocompatibitliy, and biodegradability of CaXs. In this review, the state‐of‐the‐art studies on GOx‐mineralized CaXs are introduced with an emphasis on their application in various biomedical fields including disease diagnosis, cancer treatment, and diabetes management. The current challenges and future perspectives of GOx‐mineralized CaXs are discussed, which is expected to promote further studies on these smart GOx‐mineralized CaXs biomaterials for practical applications.

## Graphical abstract

In this review, the state‐of‐the‐art studies on glucose oxidase (GOx)‐mineralized calcium‐based biomaterials (CaXs) are introduced with an emphasis on their application in various biomedical fields including disease diagnosis, cancer treatment, and diabetes management. The current challenges and future perspectives of GOx‐mineralized CaXs are discussed, which is expected to promote further studies on these smart GOx‐mineralized CaXs biomaterials for practical applications.

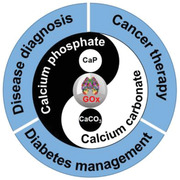

## 1 INTRODUCTION

Glucose oxidase (GOx) is a naturally sourced oxido‐reductase produced by various funguses, which can specially catalyze the oxidization of glucose to produce gluconic acid and hydrogen peroxide (H_2_O_2_) under aerobic condition.^[^ ^1^ ^]^ This fascinating catalysis has been widely applied in the biomedical fields especially these diseases related to abnormal glucose metabolism, such as cancer and diabetes.^[^ ^2^ ^]^ For instance, the GOx catalysis provides a promising non‐invasive starvation therapy to combat cancer by exhausting glucose,^[^ ^3^ ^]^ since glucose nutrients is an important energy and carbon source for tumor cells survival and proliferation.^[^ ^4^ ^]^ Moreover, this catalytic process elevates the acidity, exacerbates the hypoxia, and increases the oxidative stress of tumor microenvironment (TME), which can be combined with other therapeutic approaches to achieve synergistic efficacy.^[^ ^5^ ^]^ As a kind of natural enzyme, GOx has good biocompatibility, non‐toxicity, and biodegradability. However, some shortcomings of GOx may hinder its further applications: (1) the intravenous injection of GOx may lead to a transient decrease of blood glucose level,^[^ ^6^ ^]^ inflammation and hematotoxicity^[^ ^7^ ^]^ since the blood contains 3−5.6 mm of glucose and this value is higher after a meal (5−7.5 mm);^[^ ^2b^ ^]^ (2) GOx was found to damage mice organs such as liver and kidney;^[^ ^7^ ^]^ (3) the poor bioavailability and rapid inactivation of GOx combined with the biological barriers resulting in poor therapeutic efficacy in vivo.^[^ ^8^ ^]^ To overcome these shortcomings, the immobilization of GOx in a nanoplatform which not only avoid the premature leakage when circulating in the body, but also accumulate in the lesion and realize an endogenous stimulus‐response release without the need of external excitation is highly desirable.

Calcium‐based biomaterials (CaXs) such as calcium phosphate (CaP) and calcium carbonate (CaCO_3_) are ubiquitous in nature, and consist of the main inorganic constituents of vertebrate bones, teeth, and shells.^[^ ^9^ ^]^ CaXs are widely used in biomedical fields (e.g. protein/drug/gene delivery, tissue engineering etc.) owing to their unique properties of bioactivity and biodegradability.^[^ ^10^ ^]^ Also, these biomaterials are essentially stable under neutral condition while can dissolve under acidic condition, making CaXs an excellent drug carrier for various biomedical applications such as cancer treatment since the pH of TME is around 6.5−6.9.^[^ ^11^ ^]^ CaXs can be absorbed by organisms or excreted from the body by dissolving into non‐toxic ions, making them safer candidates for clinical applications. In addition, CaXs are easily obtained by a facile biomimetic mineralization process by utilizing biomolecules (such as proteins, enzymes, nucleic acids, and polypeptides) as biotemplates.^[^ ^12^ ^]^ Through this process, these biomolecules are encapsulated in CaXs rather than adsorbed on the surface of it. Importantly, biomineralization is reported to maintain the bioactivity of these biomolecules under physiological conditions and achieve a sustained drug release behavior.^[^ ^12^, ^13^ ^]^ Hence, the encapsulation of GOx in CaXs through a biomineralization process is expected to improve the stability, bioavailability and therapeutic efficacy against many diseases including cancer through its activatable release behavior, and reduce the side effects to normal tissues.

**FIGURE 1:**
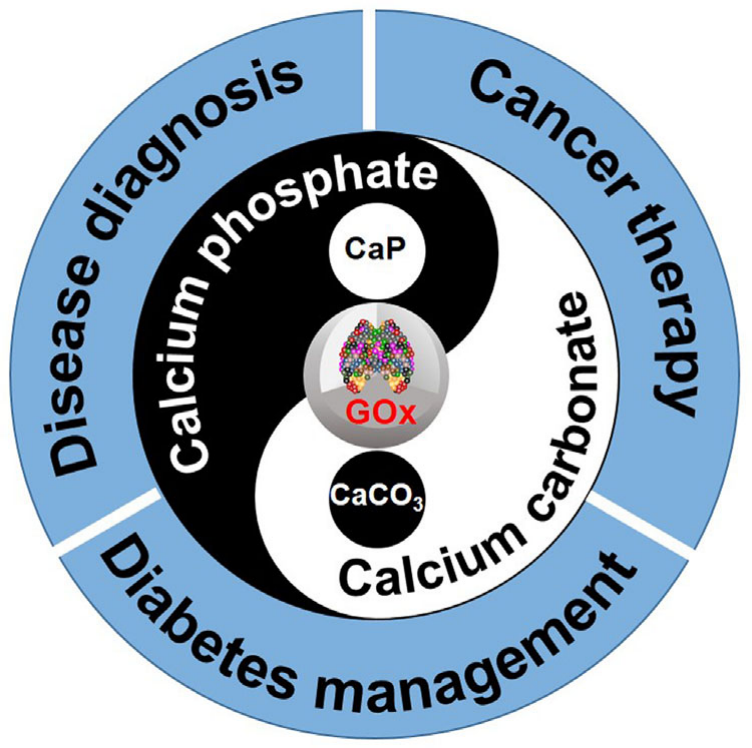
The main biomedical applications of GOx‐mineralized CaP and CaCO_3_.

Although some relevant review papers have summarized the biomedical applications of GOx and CaXs,^[^ ^2^, ^5^, ^14^ ^]^ there still remain some limitations. These publications have reviewed the CaXs design and applications in nanomedicine and individual reviews briefly introduced the utilization of protein as biotemplate to obtain CaXs,^[^ ^14c^ ^]^ while the GOx‐related reviews often focused on the topic of cancer detection and synergistic therapies.^[^ ^2^, ^5^ ^]^ Considering the great potential and research fever of GOx in biomedical fields especially cancer and diabetes, and the unique properties of CaXs that can conquer the shortcomings of GOx in vivo to improve therapeutic efficacy, a review concentrates on the GOx‐mineralized CaXs and their application in biomedical fields is urgently needed. So far, the CaXs that have been reported including CaP, CaCO_3_, calcium silicate (CaSiO_3_), calcium fluoride (CaF_2_), calcium sulfide (CaS), and calcium peroxide (CaO_2_), while almost the studies related to mineralization of GOx are focus on CaP and CaCO_3_. This may be due to the essential biocompatibitliy and bioresorbable, good stability in the air, and easily to prepare in aqueous phase of CaP and CaCO_3_ than other CaXs. For instance, the CaS and CaO_2_ are unstable and their synthesis often involve organic solvents such as ethanol,^[^ ^15^ ^]^ which may impair the activity of GOx. On the other hand, GOx‐mineralized CaXs combine the advantages of both GOx (natural enzyme with specific catalytic performance against glucose) and CaXs (excellent biocompatibility and pH‐sensitive biodegradability), while CaXs can maintain the bioactivity of GOx from harsh conditions and reduce the side effects of GOx on normal tissues. Therefore, compared with CaXs prepared by other methods, GOx‐mineralized CaXs is more potential in biomedical fields. In this review, we focus on the recent advances of GOx‐mineralized CaXs including CaP and CaCO_3_ with an emphasis on their application in various biomedical fields including disease diagnosis, cancer treatment, and diabetes management (Figure 1). Then we discuss the current challenges and future perspectives and hope to stimulate extensive further studies on these smart GOx‐mineralized CaXs biomaterials, promoting their practical applications.

## 2 BIOMIMETIC MINERALIZATION

Since GOx‐mineralized CaXs is mainly obtained via biomimetic mineralization strategy, understanding the mineralization process is essential for the design and construction of novel multifunctional nanoplatforms. Biomineralization is a widespread phenomenon in nature and serves as an inspiration for scientists for decades. Living organisms use biomolecules as matrix or template for the deposition and growth of biominerals such as CaP and CaCO_3_ to form bone, teeth, nacre, and shells,^[^ ^9^, ^16^ ^]^ providing mechanical supporting or defense system for them. During this process, generally, the negative charged surface of biomolecules provide the nucleation sites by absorbing Ca^2+^ ions to increase the local supersaturation, which inducing CaP or CaCO_3_ minerals formation in situ.^[^ ^17^ ^]^ Through this process, the biomolecules are encapsulated in biominerals, which can improve the stability and biocompatibility of encapsulated biomolecules, and achieve a pH‐responsive release behavior due to the acid response degradation performance of biominerals like CaP and CaCO_3_.

To date, various biomolecules including enzymes (e.g GOx, catalase), nucleic acid, proteins and protein products, hyaluronic acid, polyamino acid, polysaccharides and so on^[^ ^10^, ^12^, ^14^, ^18^ ^]^ are explored as templates for the biomimetic mineralization of CaP and CaCO_3_. These biomolecules serve as accelerator and regulator for the formation of biominerals, control the physicochemical properties (including structure, size, morphology and chemical composition) of the formed biominerals to achieve fascinating properties.^[^ ^12^, ^19^ ^]^ In turn, the biocompatible and biodegradable biominerals serve as stealth clothes to protect encapsulated biomolecules from the host immune system and reduce their toxicity. Moreover, it is easy to incorporate foreign ions (e.g. Mn^2+^, Cu^2+^, Fe^2+^/Fe^3+^, Mg^2+^, Zn^2+^, Sr^2+^, etc.) to the amorphous minerals during the biomineralization process, which may endow them with multifunctions in imaging and therapy.^[^ ^14^, ^20^ ^]^ Hence, biomineralization of GOx by these biominerals like CaP and CaCO_3_ is promising, which is expected not only improve the stability and biocompatibility of GOx but also reduce its side effects to normal tissues due to the activatable release behavior of biominerals. Moreover, by adding the foreign ions during the mineralization process, it is easily to construct multifunctional GOx nanoplatforms for diagnosis and treatment. Herein, considering that the synthetic process of GOx‐mineralized CaXs is very simple, which mixing GOx, calcium salt, carbonate or phosphate in aqueous medium under room temperature or 37°C, we will not describe more details about it. The construction of various GOx‐mineralized CaXs nanomedicine will be introduced in detail by the examples in Sections 3.2 and 3.3.

## 3 GOx‐MINERALIZED CAXS FOR BIOMEDICAL APPLICATIONS

### 3.1 Disease diagnosis

The application of CaXs mineralized GOx for the diagnosis of diseases is rarely reported. The only attempt was employing CaXs‐mineralized GOx to detect intracellular glucose level, since the disordered glucose metabolism is related to some diseases especially diabetes mellitus and cancer. The fasting blood containing about 3−5.6 mm of glucose and this value increased to 5−7.5 mm for the postprandial blood.^[^ ^2b^ ^]^ Glucose concentrations below 3 mm or exceed 7 mm may be considered pathological changes. For diabetes, the increased blood glucose level is related to some serious complications such as neuropathy, coma, seizures, kidney failure, vision impairment, and cardiovascular disease.^[^ ^21^ ^]^ Hence, the timely and accurately detection of blood glucose level is useful for the diagnose and treatment monitoring of the diabetes. For cancer, the cells often upregulate glycolysis to supply adenosine triphosphate (ATP) and carbon source to promote fast proliferation, resulting in significantly increased glucose consumption compared with normal cells, and this metabolic alteration is more pronounced in aggressive tumors.^[^ ^4^, ^22^ ^]^ Based on this feature, the glucose analogue tracer 18‐fluorodeoxyglucose is utilized to reflect the abnormal increased glucose uptake by positron emission tomography (PET) imaging, which can identify near 90% of primary and metastatic lesions in the clinical practice.^[^ ^23^ ^]^ The accurate detection of intracellular glucose level to reveal cancer cell metabolism is critical for effective treatment of cancer.

Chen et al. developed a size controllable GOx encapsulated amorphous calcium phosphate (ACP) composites for the detection of intracellular glucose.^[^ ^24^ ^]^ The biocompatible ACP‐GOx NPs showed high chemical and thermal stability after immobilization. Two types of cancer cells (SNU‐354 and H1299) and one normal cell (L02) with different metabolism rates were chosen as models, and the 2′,7′‐dichlorodihydrofluorescein (DCFH) dye labeled ACP‐GOx NPs were used to monitor the glucose concentration in these cells. When ACP‐GOx uptook by the cells, GOx could catalyze glucose decomposition to generate H_2_O_2_ which further oxidized DCFH to a fluorescent one. As expected, both SNU‐354 and H1299 cells showed higher fluorescence intensity than that of L02 cell due to the higher glucose levels. As a comparison, the conventional cell lysis method also used to measure the glucose concentration in these cells, the highest glucose concentration was detected in H1299 cells, and the normal cells L02 was the lowest. The linear relationship of glucose concentration with ACP‐GOx NPs fluorescence intensity making ACP‐GOx NPs a good biosenser to measure the intracellular glucose levels.

Although GOx are widely used to diagnose diabetes mellitus and cancer,^[^ ^2a,2b^ ^]^ there are few attempts that used GOx‐mineralized CaXs as biosensor to detect the blood glucose level or the intracellular glucose concentration. The possible reason is there have been numerous studies on glucose detection of diabetes and cancer with good detection sensitivity and detection limit, and people can even measure glucose levels using commercial glucometers by themselves. For cancer, PET imaging is widely used to diagnose the lesions with abnormal glucose metabolism in clinical practice as a golden standard, although PET is difficult to resolve lesions less than 0.8 cm^3^.^[^ ^23^ ^]^ Besides, numerous sensors and methodologies were developed to determine glucose concentration in real‐time, including electrochemistry, colorimetry, conductometry, and fluorescent spectroscopy.^[^ ^2^, ^25^ ^]^ CaXs‐mineralized GOx can effectively improve the stability of GOx while its timeliness and detection limit are difficult to reach or go beyond existing methods, since CaXs is relatively stable and need external stimulus like acidic condition to decompose it and release immobilized GOx. In this view, CaXs mineralized GOx is more suitable for the treatment of diabetes mellitus and cancer with long‐term sustained release properties. In addition, GOx has been widely utilized to construct a serious of oxygen‐, pH‐, and H_2_O_2_‐based biosenors to detect various biomarkers (e.g. α‐fetoprotein, prostate specific antigen, DNA, MicroRNA, immunoglobulin G etc) besides glucose.^[^ ^2^, ^26^ ^]^ Hence, the potential of GOx‐mineralized CaXs‐based biosensors in detecting these biomarkers remain to be explored.

### 3.2 Cancer therapy

Cancer is one of the deadliest diseases worldwide with high morbidity and mortality. According to the GLOBOCAN 2020 estimates on 36 cancers in 185 countries, the new cancer cases and cancer deaths in 2020 are 19.3 and 10.0 million, respectively, and the global cancer burden will reach 28.4 million cases by 2040.^[^ ^27^ ^]^ The effective cancer treatments are critical for improving life quality and prolonging patient survival. Although various strategies such as photothermal therapy (PTT), gene therapy, photodynamic therapy (PDT), immunotherapy, and gas therapy have been quickly developed, chemotherapy, radiotherapy, and surgery are still the mainstream treatments in current clinical.^[^ ^28^ ^]^ Cancer is featured with reprogrammed cellular metabolism, especially the glucose metabolism, and various types of tumors rely on glycolysis for energy production and provide them with a series of growth advantages.^[^ ^29^ ^]^ Starvation therapy based on blocking glucose metabolism has aroused a research boom in recent years, making GOx a “star” therapeutic enzyme. In this section, the application of CaP‐ and CaCO_3_‐mineralized GOx nanomaterials for cancer therapy (Table 1) will be discussed in detail.

As aforementioned, there are several similar properties between CaP and CaCO_3_, such as both of them possess excellent bioactivity, biocompatibitlity, and easily obtained by a simple biomineralization strategy. They can encapsulate GOx during the biomimetic mineralization process, improve the stability, biocompatibitlity, and bioavailability of GOx. Because of their acid‐responsive degradability and bioabsorbability, they can selectively deliver GOx to the mild acidic TME with minimal side effects. Under acidic condition, CaP will dissolve into Ca^2+^ and PO_4_^3−^, while CaCO_3_ degrades to release Ca^2+^ and CO_3_^2−^. Different kinds of anions give them with different functions. For instance, PO_4_^3−^ is reported to restore the activity of adenosine kinase (ADK), which reduces the accumulation of adenosine (Ado) in tumor tissue and relieve the Ado‐mediated immunosuppression, resulting in enhanced antitumor immune response.^[^ ^30^ ^]^ High concentration of CO_3_^2−^ in aqueous medium will release CO_2_ bubble, which have been widely used in ultrasonic therapy and ultrasonic imaging.^[^ ^31^ ^]^ Hence, it is necessary to review the recent advance of CaP‐mineralized GOx and CaCO_3_‐mineralized GOx in cancer therapy to stimulate more interest on them.

#### 3.2.1 GOx‐mineralized CaP

GOx possesses great potential in combating cancer due to its fascinating catalysis against glucose, while the risks of system toxicity of GOx and its inherent instability under physiological conditions that contain many hydrolases hinder its applications in vivo. In nature, the vertebrates use collagen as matrix for the deposition and growth of CaP minerals to form bone and teeth.^[^ ^16a,16b^ ^]^ Inspired by this phenomenon, GOx was employed as template for the biomimetic mineralization of CaP.^[^ ^20^, ^32^ ^]^ CaP holds outstanding bioactivity and innate pH‐dependent solubility, which insures the relative stability of GOx‐CaP complex under neutral condition but can dissociate in acidic condition to release GOx due to the dissolution of CaP. In this way, the highly biocompatible and nontoxic CaP is regarded as a safe candidate for the delivery of GOx to the acidic TME.

**FIGURE 2:**
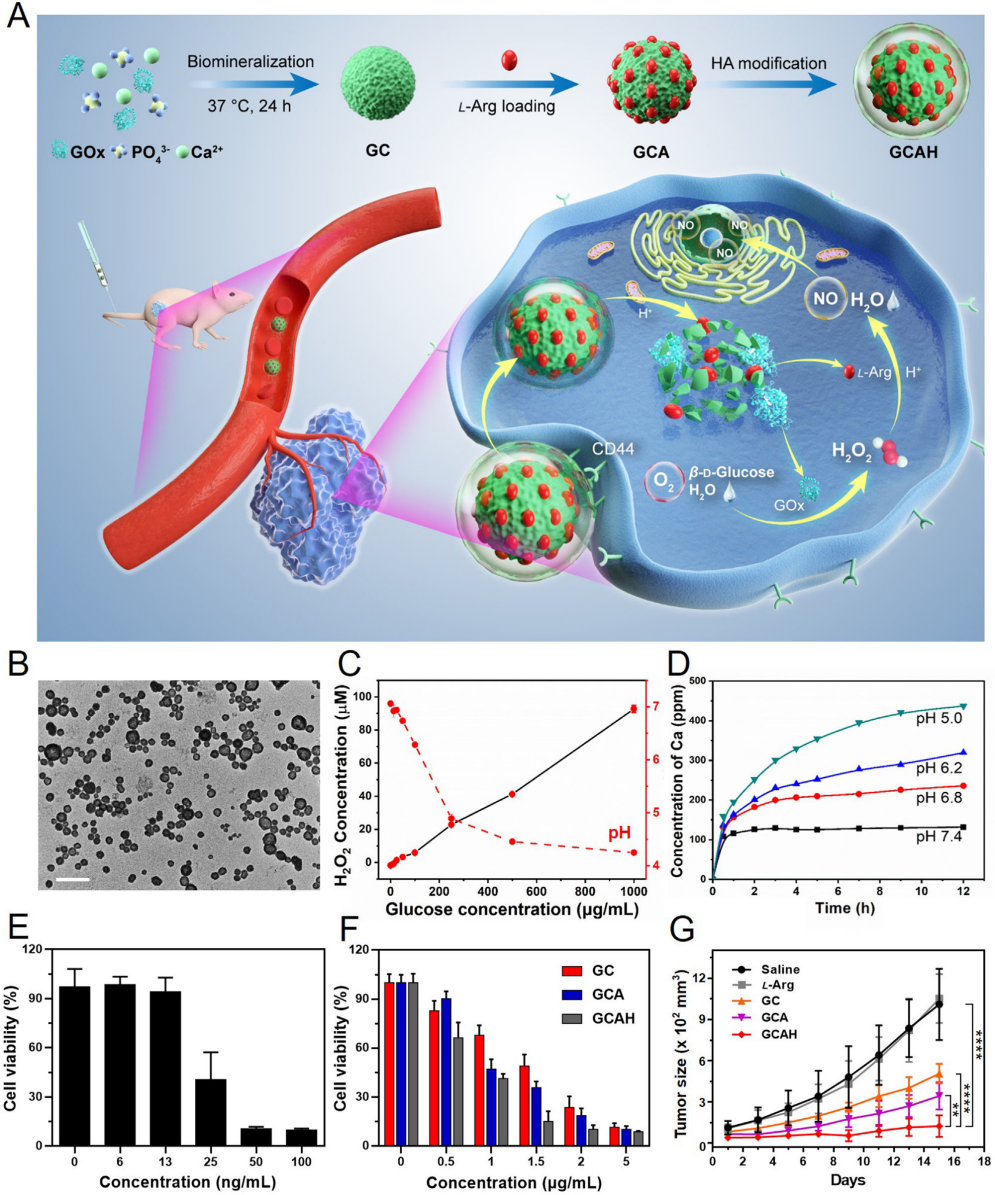
Synthesis, characterization, and antitumor activity of GCAH. (A) Schematic illustration of the synthetic route of GCAH and its application for TME‐activated synergistic starvation/gas therapy. (B) TEM image of GC, scale bar is 400 nm. (C) H_2_O_2_ generation and (D) pH variation after incubating GC with different concentration of glucose solution. (E,F) Cell viability after incubating with (E) GOx, (F) GC, GCA, and GCAH for 12 h. (G) Tumor size variation during the treatment. Reproduced with permission.^[^ ^32^ ^]^ Copyright 2021, Wiley‐VCH.

With this regard, our group has constructed a tumor targeted and TME‐activated nanoplatform GCAH for effective cancer synergistic therapy, of which the glucose nutrients were exhausted by GOx catalysis and the generated H_2_O_2_ would oxidize *L*‐Arginine (*L*‐Arg) into NO for enhanced gas therapy (Figure 2A).^[^ ^32^ ^]^ The uniform and well‐defined spherical CaP carrier was obtained by a facile one step biomineralization method using GOx as biotemplate in the glucose‐free dulbecco's modified eagle medium (DMEM) (Figure 2B). The DMEM containing 200.0 mg L^–1^ of calcium chloride (CaCl_2_, 1.8 mm), which is close to the Ca^2+^ concentration in human blood plasma (2.5 mm).^[^ ^33^ ^]^ Under this concentration, the Ca^2+^ ions could be absorbed by GOx to provide initial nucleation sites since the GOx (isoelectric point is around 4.9) was negative charged in DMEM. After 10 mm of CaCl_2_ aqueous solution was added in the reaction system, the CaP mineralization would be triggered by the excess Ca^2+^ ions and the phosphorus source from DMEM that containing 125.0 mg L^–1^ of sodium phosphate monobasic (NaH_2_PO_4_•H_2_O, 0.9 mm) to obtain GOx‐CaP complex (named as GC).

The biomineralization process had little influence on the catalytic activity of GOx since a large amount of H_2_O_2_ was generated when incubated GC with glucose solution (Figure 2C), and the pH value was dramatic dropped (Figure 2C) which could trigger the degradation of GC (Figure 2D) to release the cargos. As expected, the mineralization of CaP could effectively improve the biocompatibility of GOx, more cells were survived after treated with GC than that of free GOx (Figure 2E,F). After *L*‐Arg loading and hyaluronic acid (HA) modification, the lowest 4T1 tumor cell viability was achieved when incubated with GCAH due to the cascade reactions of GOx and *L*‐Arg and HA‐mediated improvement of cellular uptake. In vivo experiments further verified the remarkable synergistic starvation/gas therapeutic efficacy on 4T1‐tumor bearing mice (Figure 2G).

**FIGURE 3:**
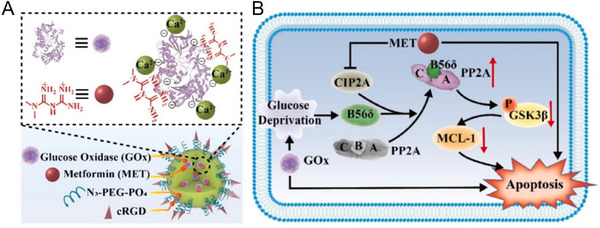
Illustration of (A) MET@CaP‐GOx‐PEG‐cRGD nanoplatform and (B) therapeutic synergism between MET and GOx‐mediated glucose depletion. Reproduced with permission.^[^ ^34^ ^]^ Copyright 2021, Elsevier.

By using the same strategy, Luo et al. constructed a biomineralized nanoplatform MET@CaP‐GOx‐PEG‐cRGD via spontaneous biomineralization of GOx and metformin (MET) in glucose‐free DMEM, and sequentially modified with polyethylene glycol (N_3_‐PEG‐PO_4_) and cRGD (Figure 3A).^[^ ^34^ ^]^ The modification of PEG and cRGD ensured the accumulation of this nanoplatform in tumor overexpressed *α*_v_ *β*_3_ integrin after systemic administration, while the acid‐responsive degradable CaP could prevent premature release and improve the GOx/MET delivery. GOx converted glucose in the tumor cytoplasm into gluconic acid and H_2_O_2_, which not only starved tumor cells and upregulated B56δ expression, but also improved the acidity and facilitated drug release. B56δ is a regulatory subunit of phosphatase 2A (PP2A) while MET is reported to relieve PP2A inhibition by downregulating the expression of cancerous PP2A inhibitor.^[^ ^35^ ^]^ Therefore, the combination of glucose depletion and MET is expected to enhance the PP2A expression and promote the dephosphorylation of downstream glycogen synthase kinase 3β (GSK3β), resulting in the inhibition of anti‐apoptotic MCL‐1 proteins and promoting tumor cell apoptosis (Figure 3B). In this study, the MET@CaP‐GOx‐PEG‐cRGD was observed to effectively inhibit the growth of two *α*_v_ *β*_3_ integrin over‐expressed tumor cell lines (HepG2 and B16F10 melanoma cells) via regulating the PP2A‐GSK3β‐MCL‐1 axis, and good cytocompatibility to the normal LO2 cells that low expressed *α*_v_ *β*_3_ integrin and GSK3β. The synergistic antitumor efficacy of this nanoplatform was also demonstrated on xenografted HepG2 tumors without side effects, which may provide new avenue for targeted tumor therapy.

Except in situ biomineralization in DMEM media, Chen et al. fabricated size controllable and dispersive nanospherical GOx‐loaded ACP composites by co‐precipitation and nano‐channel extrusion method.^[^ ^24^ ^]^ The size of ACP‐GOx nanoparticles (NPs) can be adjusted by simply change the pore size of the polycarbonate membrane due to the plasticity of ACP (the hardness of ACP is less than half of its crystal form). ACP‐GOx NPs with average diameters of 382, 221, and 113 nm were obtained by extrudation through polycarbonate membranes with 400, 200, and 100 nm pore, respectively. It seems that the extrusion process is more like the multi‐stage centrifugation which can separate NPs with different size ranges by changing the speed of rotation, and it is easier to operate and realize mass production than that of nano‐channel extrusion method. In another case, considering that alginates (Alg) can partially alleviate the aggregation of CaP due to the affinity between Ca^2+^ and –COOH of Alg, Wu et al. immobilized GOx on Alg through amidation first, then the GOx‐Alg was mixed with CaCl_2_, curcumin (Cur), obatoclax (Obx), and the mineralization was stimulated by adding sodium polyphosphate under stirring.^[^ ^36^ ^]^ GOx‐Alg was demonstrated to control the irregular aggregation of CaP, and spherical GOx‐Alg@CaP/Cur‐Obx NPs with size of 204 nm was obtained. The Cur augmented mitochondrial Ca^2+^ overload that induced by Alg@CaP, resulted in mitochondrial dysfunction to enhance the therapeutic efficacy of GOx. Obx is an autophagy inhibitor, which inhibited cancer cell autophagy, further boosted the nutrient starvation‐dominated cancer therapy.

#### 3.2.2 Ion doped GOx‐mineralized CaP

**FIGURE 4:**
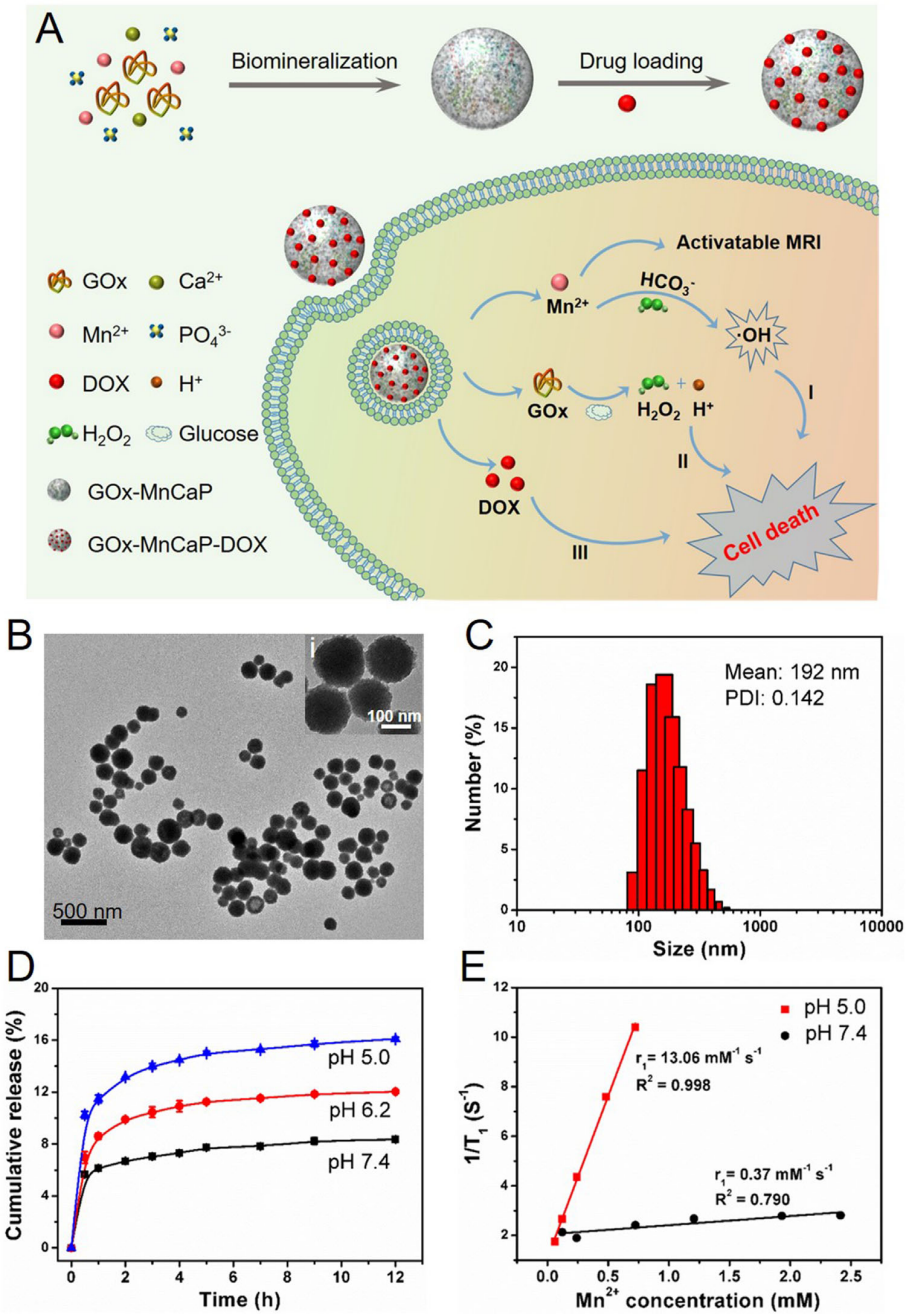
Synthesis, characterization, and application of GOx‐MnCaP‐DOX. (A) Schematic illustration of the synthesis process of GOx‐MnCaP‐DOX and its application for MRI‐monitored cooperative cancer therapy. (B) Representative TEM image and (C) hydrodynamic size distribution of GOx‐MnCaP NPs. (D) Release profile of DOX from GOx@MnCaP‐DOX in PBS buffer. (E) 1/*T*_1_ of GOx‐MnCaP released PBS media as a function of Mn^2+^ concentration. Reproduced with permission.^[^ ^20a^ ^]^ Copyright 2019, American Chemical Society.

The amorphous minerals like ACP are ready to accept the incorporation of foreign ions, which can regulate the minerals’ composition, structure and solubility, even endow them with unexpected properties. For instance, the lanthanide (e.g. Eu^3+^, Tb^3+^, Nd^3+^)‐doped CaP have excellent luminescent properties, while the magnetic ions (e.g. Fe^2+^/Fe^3+^, Mn^2+^, Gd^3+^) doping renders CaP for magnetic resonance imaging (MRI) and magnetic hyperthermia therapy.^[^ ^14b^ ^]^ Inspired by the previous studies, our group developed GOx‐MnCaP NPs by mineralizing GOx in sugar‐free DMEM with doping of Mn^2+^, and then served as carrier for an anticancer drug doxorubicin (DOX) (Figure 4A).^[^ ^20a^ ^]^ The monodispersed GOx‐MnCaP NPs with spherical morphology and average hydrodynamic size of 192 nm were obtained (Figure 4B,C). When internalized by the tumor cells, GOx‐MnCaP‐DOX would degrade to release GOx, Mn^2+^, and DOX (Figure 4D) under acidic TME. GOx could effectively delete the intratumoral glucose to starve tumor cells and elevate H_2_O_2_ level to enhance oxidative stress. On the other hand, the released paramagnetic Mn^2+^ played two roles: (1) acted as MRI contrast agent due to the pH‐responsive contrast effect (Figure 4E), (2) served as Fenton‐like reagent that converted H_2_O_2_ to highly toxic hydroxyl radicals (•OH). Combining with the anticancer drug DOX, GOx‐MnCaP‐DOX was demonstrated high antitumor efficacy via the orchestrated cooperative cancer starvation therapy/chemodynamic therapy (CDT)/chemotherapy after intratumoral administration. Although the biodegradable GOx‐MnCaP‐DOX nanotheranostics with good antitumor effect holds clinical translation potential, the intratumoral administration due to the lack of active targeting and large size of it may hinder its further application when come to the deep tissue tumors.

**FIGURE 5:**
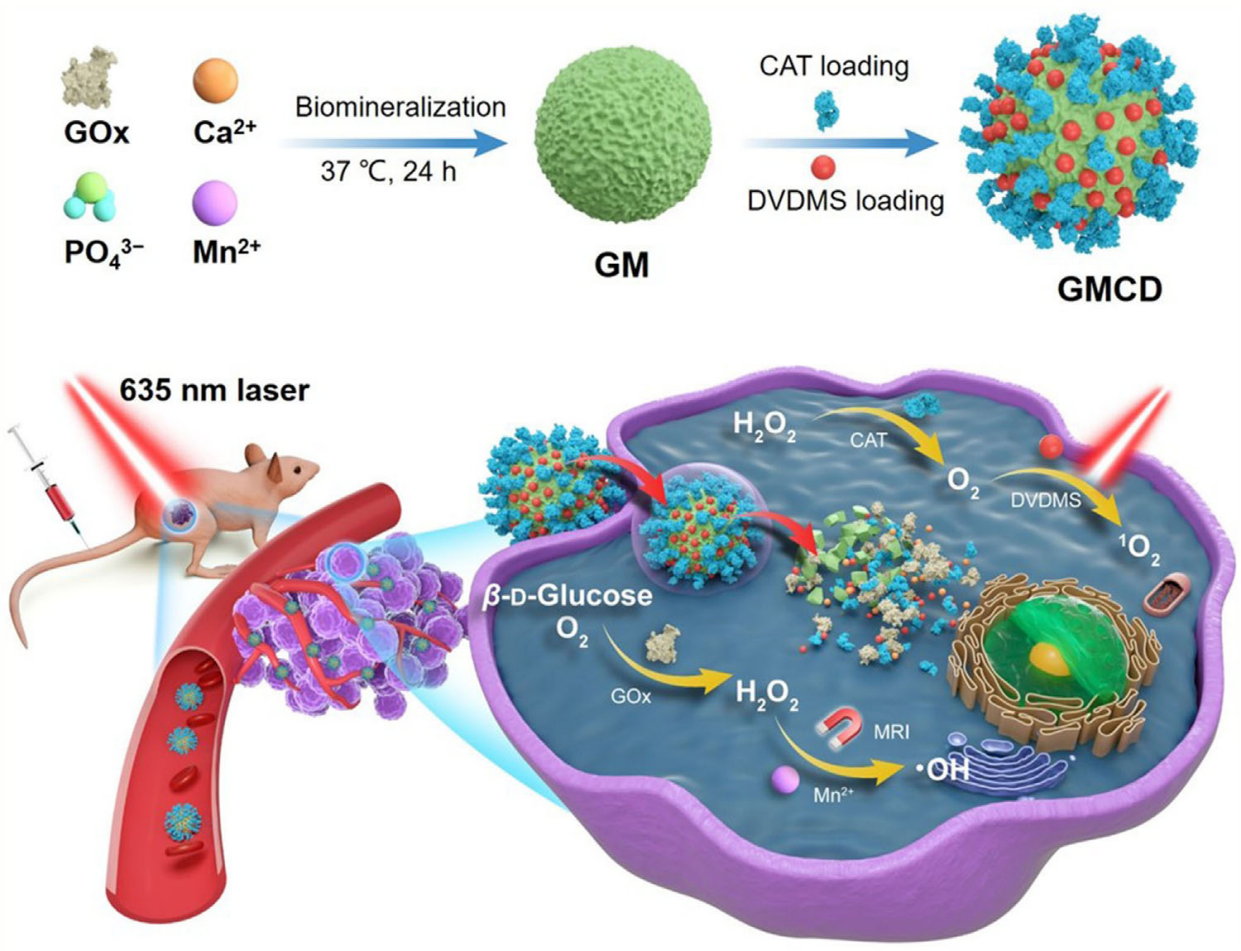
Schematic illustration of the preparation of GMCD and its application for TME‐activatable cascade catalytic reactions‐enhanced PDT. Reproduced with permission.^[^ ^20b^ ^]^ Copyright 2021, Wiley‐VCH.

Sinoporphyrin sodium (DVDMS) is a kind of porphyrin‐based photosensitizers, which is found to effectively accumulate in solid tumor^[^ ^37^ ^]^ and can coordinate with metal ions like Mn^2+^ to form nanoassemblies for cancer phototherapy.^[^ ^38^ ^]^ Hence, the GOx‐MnCaP NPs is expected as a good carrier for DVDMS, which may achieve high drug loading capacity due to the interaction between Mn^2+^ and DVDMS and the loaded DVDMS may endow tumor targeting activity for the NPs. In Fu's another work, the GOx‐MnCaP was employed as a carrier for co‐loading of DVDMS and catalase (CAT) to construct a biodegradable TME‐specific activatable nanoplaform (named as GMCD) for cascade catalytic reactions‐augmented PDT (Figure 5).^[^ ^20b^ ^]^ Solid tumors often suffer from hypoxia,^[^ ^39^ ^]^ while GOx catalysis will aggravate hypoxia, damaging the efficacy of PDT which relied on O_2_ to generate singlet oxygen (^1^O_2_). CAT is co‐delivered to alleviate hypoxia by catalytic decomposition of both the endogenous H_2_O_2_ and GOx catalysis generated H_2_O_2_ to O_2_, which can not only replenish O_2_ for GOx catalysis but also improve production of ^1^O_2_ to enhance PDT efficacy. As expected, GOx‐MnCaP showed high drug loading capacity (97.7%) against DVDMS. Importantly, the quenched fluorescence signal of loaded DVDMS was recovered in tumor site due to the pH‐responsive degradation of GMCD, resulting in an “off to on” fluorescence transduction which can visualize the in vivo DVDMS release by fluorescence imaging. After intravenous administration, GMCD was effectively accumulated in the tumor and achieved a sustained DVDMS release behavior (up to 144 h). After a single injection of GMCD, the 4T1 tumor growth was completely inhibited or eliminated, making GMCD a promising candidate in clinical cancer treatment.

**FIGURE 6:**
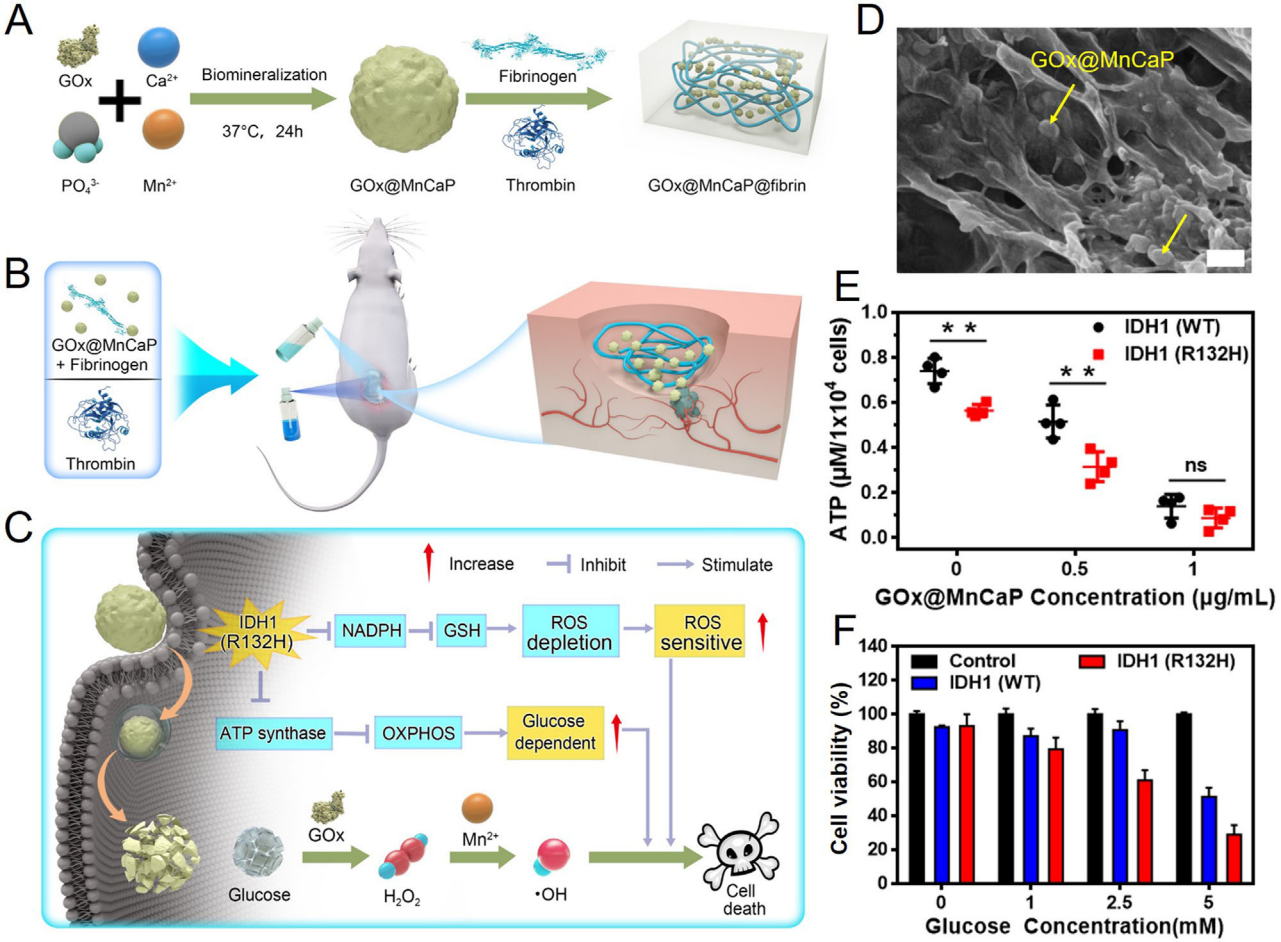
Synthesis, characterization, and antitumor activity of GOx@MnCaP@fibrin gel. (A–C) Schematic illustration of the synthetic route of GOx@MnCaP@fibrin gel and its application for the post‐surgical treatment of IDH1 (R132H) glioma: (A) the preparation process of GOx@MnCaP@fibrin gel; (B) the mixture of GOx@MnCaP and fibrinogen was sprayed with thrombin solution to prepare the gel in the post‐surgical cavity; (C) GOx@MnCaP@fibrin gel killed the residual IDH1 (R132H) cells. (D) Representative SEM image of GOx@MnCaP@fibrin gel. Scale bar: 1 μm. (E) ATP level in IDH1 (WT) and IDH1 (R132H) cells after treated with 0−1 μg mL^–1^ of GOx@MnCaP. (F) Viability of IDH1 (WT) and IDH1 (R132H) cell after treated with 0.5 μg mL^–1^ of GOx@MnCaP with 0−5 mm of glucose. Reproduced with permission.^[^ ^40^ ^]^ Copyright 2022, Wiley‐VCH.

Besides, the GOx@MnCaP NPs were encapsulated into a fibrin gel that composed of fibrinogen and thrombin, and sprayed in the post‐operative isocitrate dehydrogenase 1 (IDH1) (R132H) glioma site to kill the residual cancer cells and prolong the mice survival (Figure 6A–C).^[^ ^40^ ^]^ IDH1 is an important enzyme in tricarboxylic acid cycle and affects the generation of nicotinamide adenine dinucleotide phosphate (NADPH), further affects the regeneration of glutathione (GSH).^[^ ^41^ ^]^ Therefore, the R132H mutated IDH1 (IDH1 (R132H)) cells are more sensitive to reactive oxygen species (ROS) due to the decrease of important intracellular antioxidants of NADPH and GSH. In addition, IDH1 (R132H) cells are more sensitive to the variation of glucose since the cells produce ATP mainly through glycolysis, which is an inefficient energy production way.^[^ ^42^ ^]^ GOx@MnCaP NPs were dispersed in fibrinogen solution first, then sprayed the mixture with thrombin solution to prepare the GOx@MnCaP@fibrin gel. As shown in Figure 6D, GOx@MnCaP NPs were observed buried in the gel. After being treated with GOx@MnCaP, the ATP level of IDH1 (R132H) cells were decreased more than that of IDH1 (WT) cells (Figure 6E), and higher cell toxicity was observed in IDH1 (R132H) cells (Figure 6F). GOx catalyzed glucose oxidation to generate H_2_O_2_, which was further converted into highly toxic •OH by Mn^2+^ to kill the cancer cells. GOx@MnCaP NPs were found effective inhibit the proliferation and migration of IDH1 (R132H) cells mainly due to the disturbing of p53 pathway activation. Since the migration and proliferation of post‐surgical residual tumor cells are responsible for tumor recurrence, the GOx@MnCaP@fibrin gel has potential to inhibit IDH1 (R132H) tumor recurrence, which was demonstrated on a subcutaneous U87 tumor surgical resection model in this study. The lowest recurrence rate (3/8) of IDH1 (R132H) U87 tumor was achieved and the survival rate of mice reached to 82.5% within 60 days, providing a good paradigm for the post‐surgical treatment of IDH1 (R132H) glioma. Considering that surgical excision is one of the main clinical treatments of cancer, while post‐surgical prognosis is critical for prolonging the lifetime of patients. The GOx@MnCaP@fibrin gel which can effectively kill the post‐surgical residual tumor cells to reduce tumor recurrence, holds potential to treat various cancers.

**FIGURE 7:**
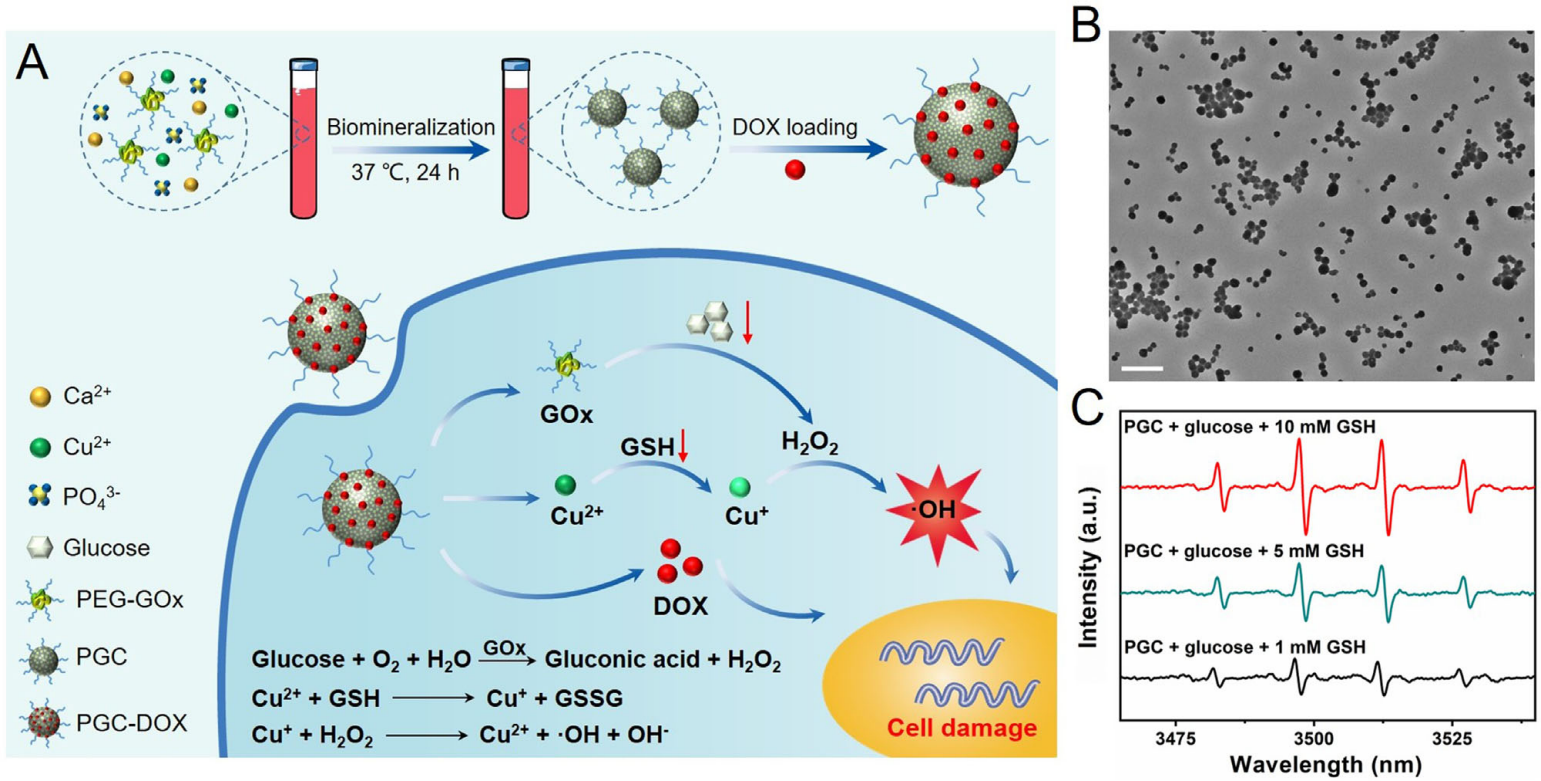
Synthesis, characterization, and application of PGC‐DOX. (A) Schematic illustration of the synthetic process and therapeutic mechanism of PGC‐DOX for sequential catalytic reactions‐induced cooperative cancer therapy. (B) Representative TEM images of PGC. (C) ESR spectra of these reaction systems. Reproduced with permission.^[^ ^20c^ ^]^ Copyright 2021, Wiley‐VCH.

Except for Mn^2+^ ions, Cu^2+^ ions were also doped in GOx‐mineralized CaP NPs, endowing the NPs with more functionalities. In a recent work, Fu et al. modified GOx with poly(ethylene glycol) (PEG) firstly, then the PEG‐GOx was used as a template to mineralize CaP in the sugar‐free DMEM with the doping of Cu^2+^ ions, named as PGC.^[^ ^20c^ ^]^ After being loaded with an anticancer drug DOX, the obtained PGC‐DOX could effectively inhibit 4T1 tumor growth via synergetic cancer starvation therapy/CDT/chmemotherapy (Figure 7A). The highly dispersed PGC with uniform size (88±17 nm) and spherical structure were obtained (Figure 7B), and PGC was observed accumulated in tumor tissue effectively may attributed to the appropriate size and the PEG modification that prolong the circulation in the body. It is well known that TME is featured by over‐expressed H_2_O_2_, low pH, over‐expressed enzymes, ATP and GSH.^[^ ^43^ ^]^ For the treatments that relied on the generation of ROS to kill cancer cells like CDT and PDT, the over‐expressed GSH (up to 10 × 10^−3^ m) in TME may weaken the therapeutic efficacy by the scavenging the generated ROS. The Cu^2+^ ions can effectively deplete intracellular GSH by converting it into oxidized glutathione (GSSG), meanwhile, Cu^2+^ can be converted to an effective Fenton's reagent, Cu^+^ ions.^[^ ^44^ ^]^ Hence, the doped Cu^2+^ ions in Fu's work were expected to improve antitumor effects by depleting the cellular antioxidant of GSH and amplifying the generation of •OH. The enhanced •OH generation with depletion of GSH was demonstrated by ESR spectra (Figure 7C). Combing the GOx‐mediated starvation therapy, Cu^2+^‐related GSH depletion and •OH generation, and DOX‐induced chemotherapy, the PGC‐DOX effectively inhibited the 4T1 tumor growth by both intratumoral and intravenous administration. As aforementioned, CaP is featured by the structural capacity which can accept many ionic substitutions, the Mn^2+^‐ and Cu^2+^‐doped GOx‐mineralized CaP for cancer treatment are successful examples and other ions doping with specific properties need further exploration.

#### 3.2.3 GOx‐mineralized CaCO_3_

CaCO_3_ is another ubiquitous and important inorganic biomineral in organisms, which has been widely used as drug carrier for various biomedical fields including cancer treatment.^[^ ^45^ ^]^ Similarly to CaP, CaCO_3_ is featured with excellent biocompatibility and pH‐sensitive biodegradability. In nature, CaCO_3_ exists as one unstable amorphous phase, two hydrated metastable forms (monohydrocalcite and CaCO_3_•6H_2_O), and three anhydrous crystalline phases (calcite, aragonite, and vaterite).^[^ ^14b^ ^]^ CaCO_3_ is relatively stable under neutral pH while it will decompose into Ca^2+^ and CO_2_ gas under acidic condition, which not only neutralizes the acidic TME but also realizes TME‐activable drug release, making it a good candidate for anticancer drug carrier. In addition, the generated CO_2_ can be used for both ultrasonic therapy and ultrasonic imaging which visualizes the treatment process.^[^ ^31^ ^]^ CaCO_3_ can be synthesized by simple, environmental friendly and low cost methods like precipitation method and gas‐diffusion method.^[^ ^10^, ^18^, ^46^ ^]^ During the preparation process, the biomolecules or drugs can incorporate into the CaCO_3_ NPs, which can maintain the activity and improve the bioavailability of them through a controlled release manner. For instance, an enzyme‐encased capsule was constructed by encapsulating GOx and insulin (ISN) in CaCO_3_ particles through a co‐precipitation process, followed by modification the NPs with tannic acid and polyethyleneimine.^[^ ^47^ ^]^ This capsule exhibited glucose responsive ISN release, which attributed to the GOx catalysis that convert glucose to gluconic acid, increased the acidity around the capsule and led to the disassembly of it to release ISN.

**FIGURE 8:**
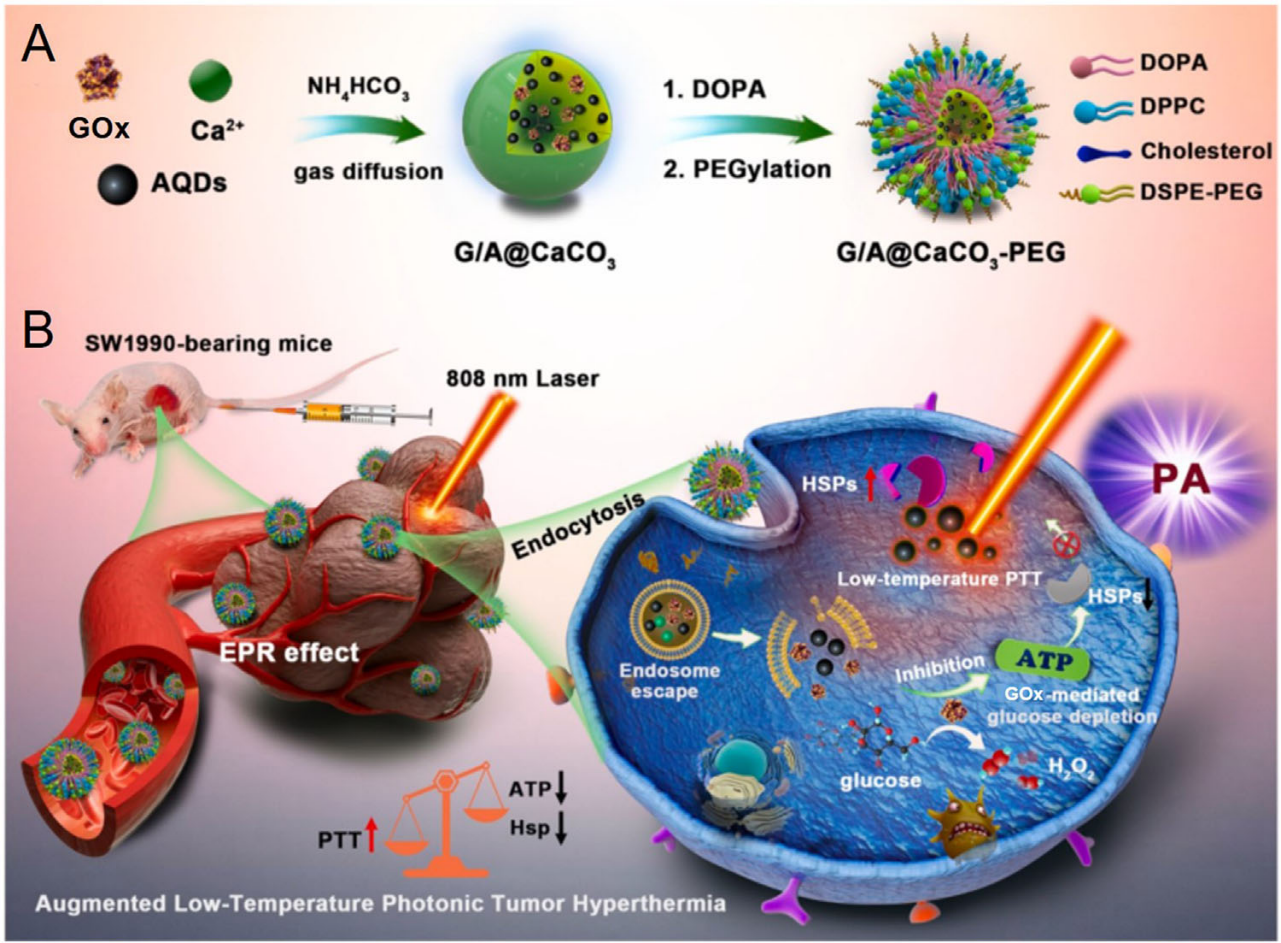
Schematic illustration of (A) the construction of G/A@CaCO_3_‐PEG nanocatalysts and (B) therapeutic mechanism against tumor. Reproduced under the terms of the Creative Commons CC BY license.^[^ ^48^ ^]^ Copyright 2022, KeAi.

Zheng et al. constructed a “non‐inhibitor involvement” nanosystem G/A@CaCO_3_‐PEG by incorporating GOx and 2D antimonene quantum dots (AQDs) into biocompatible CaCO_3_ NPs for enhanced PTT.^[^ ^48^ ^]^ As shown in Figure 8A, the G/A@CaCO_3_ was prepared by a gas‐diffusion method and modified with lipid bilayers and PEG to prolong blood circulation. As illustrated in Figure 8B, the GOx catalysis effectively exhausted glucose to block energy supply, and downregulated the ATP‐dependent heat shock protein (HSP) expression. HSP, including HSP70 and HSP90, can awake the inherent defense mechanism of tumor cells under hyperthermia‐based treatments, is important for the tumor cells’ thermoresistance.^[^ ^49^ ^]^ Hence, the restriction of ATP supply by GOx could reverse the thermoresistance of tumor cells, augmenting the therapeutic efficacy of photothermal hyperthermia at low temperature (about 43°C) induced by 2D AQDs upon 808 nm laser irradiation. The G/A@CaCO_3_‐PEG system was found to efficiently suppress the growth of SW1990 xenograft tumor without systemic toxicity, providing an inhibitor‐free strategy to overcome the tumor thermoresistance. In this study, the organic solvents including ethanol and chloroform was utilized in the preparation process, and the final product G/A@CaCO_3_‐PEG was re‐dispersed in ethanol for use. Although the authors claimed that G/A@CaCO_3_‐PEG retained sufficient enzymatic activity, it is not environment friendly and the potential effect of these organic solvents should not be ignored.

**FIGURE 9:**
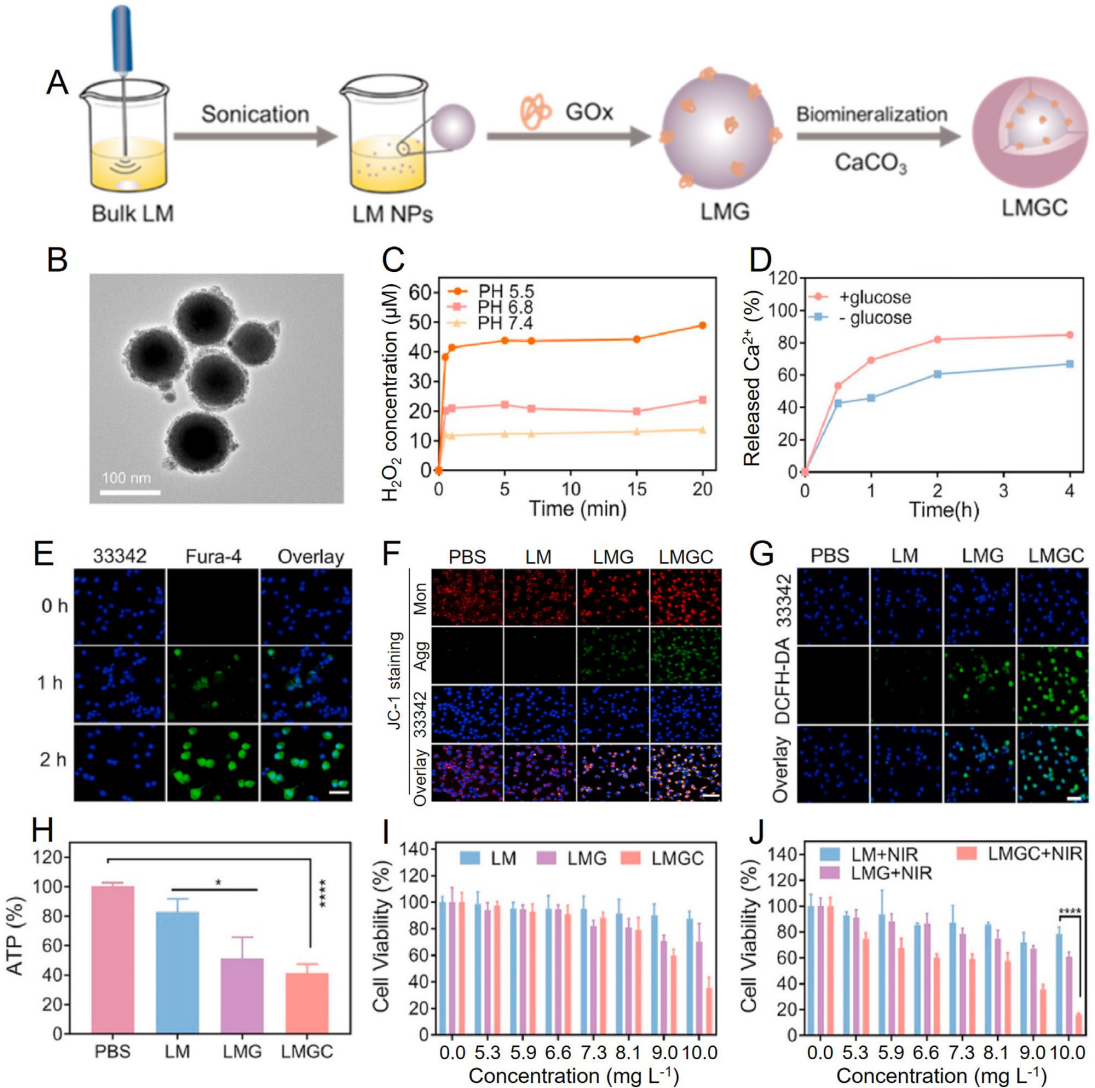
Synthesis, characterization, and anticancer activity of LMGC. (A) Illustration of the fabrication process of LMGC. (B) TEM image of LMGC NPs. Scale bar, 100 nm. (C) H_2_O_2_ generation in LMGC treated glucose solution. (D) Ca^2+^ release profile after incubation LMGC with or without glucose. (E) Intracellular Ca^2+^ level of CT26 cells treated by LMGC for different time. Scale bar, 20 μm. (F) Mitochondrial membrane potential changes of CT26 cells after different treatments. Agg, aggregation; Mon, monomer. Scale bar, 100 μm. (G) Intracellular ROS level of CT26 cells after different treatments. Scale bar, 20 μm. (H) ATP level in CT26 cells after different treatments. (I,J) CT26 cell viability after treated with LM, LMG, and LMGC with or without 808 nm laser irradiation. *p < 0.05, ****p < 0.0001. Reproduced with permission.^[^ ^50^ ^]^ Copyright 2022, Elsevier.

Similarly, Zhang et al. developed a TME‐responsive nanosystem (named LMGC) with glycolysis and mitochondrial metabolism inhibition for enhanced PTT.^[^ ^50^ ^]^ As shown in Figure 9A, the liquid metal (LM) drops with mPEG‐SH were sonicated to prepare LM NPs firstly. Then, GOx was loaded by LM NPs, and the obtained LM‐GOx (LMG) was mixed with CaCl_2_ followed by adding Na_2_CO_3_ dropwise to induce the mineralization of CaCO_3_. The LMG@CaCO_3_ (LMGC) was further modified with PEG‐PAsp to improve its dispersibility. The LMGC exhibited spherical structure (Figure 9B), and the highest H_2_O_2_ generation was achieved in LMCG treated glucose solution at pH 5.5 (Figure 9C). In addition, the LMGC triggered glucose oxidation accompanied by the generation of gluconic acid, which could amplify the acidity of TME and accelerate the decomposition of CaCO_3_ to release more Ca^2+^ ions (Figure 9D). The accumulation of Ca^2+^ in CT26 cells was observed by using a green Ca^2+^ fluorescence probe (Figure 9E), and the released Ca^2+^ caused mitochondrial dysfunction detected by an intracellular Ca^2+^ indicator (Figure 9F). On one hand, LMGC catalyzed glucose oxidation to inhibit glycolysis accompanied with H_2_O_2_ generation to elevate the oxidative stress. On the other hand, the released Ca^2+^ could disturb mitochondrial ion homeostasis and damage mitochondria, which not only amplified intracellular oxidative stress (Figure 9G), but also reduced ATP production (Figure 9H). The cut off ATP supply by glycolysis inhibition and mitochondrial dyfunction could effectively reduce the cytoprotection of ATP‐dependent HSP, resulting in improved PTT therapeutic effect (Figure 9I,J).

**FIGURE 10:**
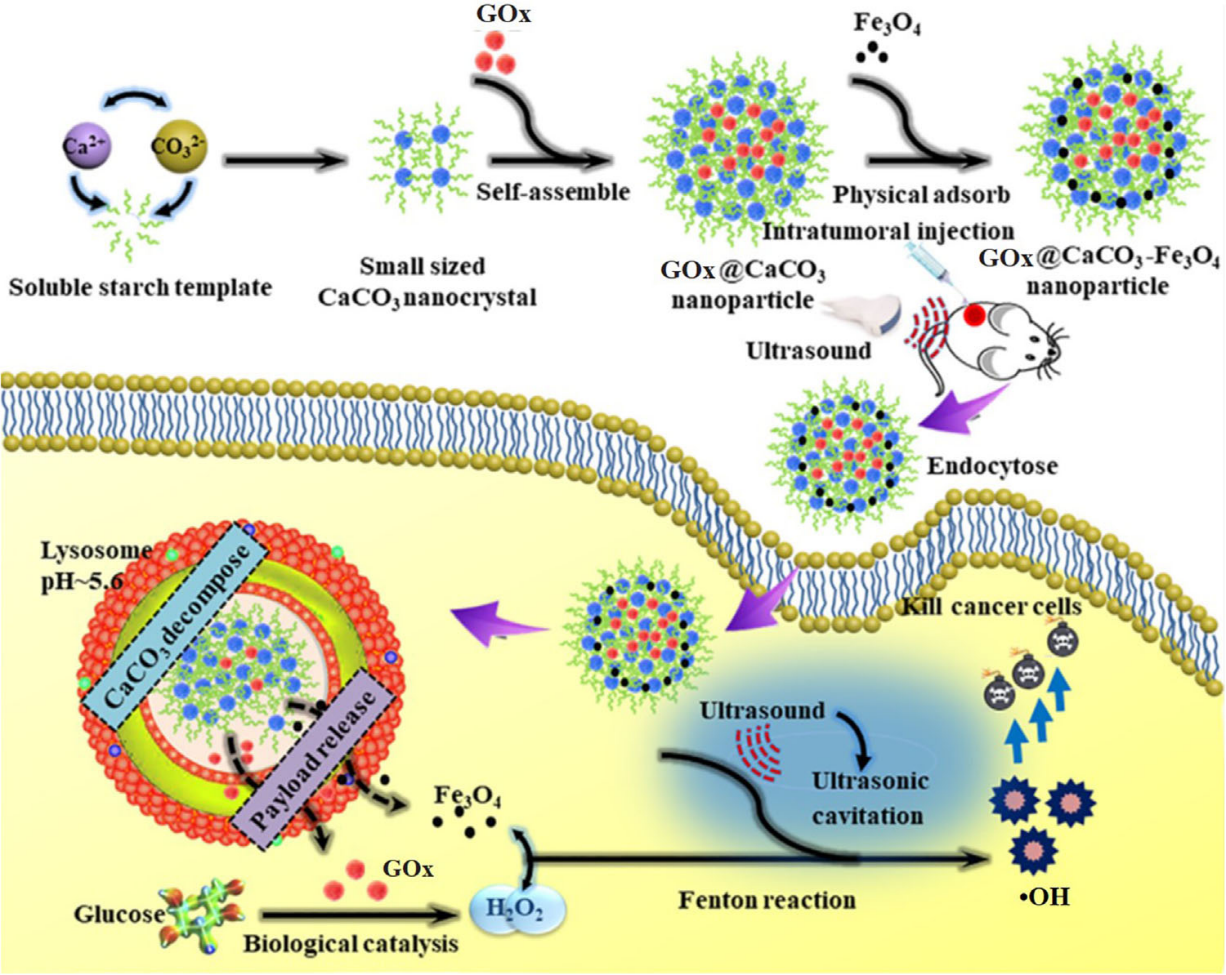
Schematic illustration of the synthetic procedure for GOx@CaCO_3_‐Fe_3_O_4_ and the sequential catalytic‐therapeutic mechanism of GOx@CaCO_3_‐Fe_3_O_4_ and ultrasound irradiation for cancer therapy. Reproduced with permission.^[^ ^51^ ^]^ Copyright 2019, Elsevier.

In another work, CaCO_3_ particles composed of stacked building units with interior cavity were prepared by precipitation method using soluble starch as template.^[^ ^51^ ^]^ As exhibited in Figure 10, the starch was mixed with CaCl_2_ solution to form small nanocrystals firstly, then Na_2_CO_3_ and GOx were added in order to trigger the self‐assembly of GOx@CaCO_3_, Fe_3_O_4_ NPs were added at last to obtain GOx@CaCO_3_‐Fe_3_O_4_ through physical adsorption. The activity of GOx was well maintained by loading it in the interior of CaCO_3_ nanocrystals, and this system could degrade to release the cargos of GOx and Fe_3_O_4_ in the acidic TME. After intratumoral injection, GOx could effectively catalyze glucose oxidation in cancer cells and produce H_2_O_2_ which was converted to •OH by Fe_3_O_4_ NPs via Fenton reaction. Ultrasound irradiation was found further improve the Fenton reaction efficacy due to the ultrasonic cavitation effect and generate abundant •OH to kill the cancer cells. Consequently, the growth of A549 xenograft tumors was completely inhibited after being treated with GOx@CaCO_3_‐Fe_3_O_4_ and ultrasound irradiation. Ultrasound has high tissue penetration depth compared with light irradiation, which has potential for the treatment of deep tissue tumors. Hence, the treatment of GOx@CaCO_3_‐Fe_3_O_4_ and ultrasound irradiation on A549 orthotopic tumors may be interesting to explore.

Recently, Song et al. reported a TME‐activatable cascade nanoreactor MG‐RNP@CaCO_3_ for Ca^2+^ ions‐enhanced CO gas therapy.^[^ ^52^ ^]^ To construct this nanoplatform, the manganese carbonyl (MnCO) and GOx were encapsulated in SiO_2_ first, then the erythroid 2‐related factor 2 (Nrf2)‐targeting Cas9/sgRNA ibonucleoprotein (RNP) was connected to the surface of SiO_2_ by disulfide linkage. Nrf2 is a regulator involved in ROS signaling pathway, the downregulation of it may impair the ROS‐mediated chemoresistance and improve therapeutic efficacy. Finally, the MnCO‐GOx‐SiO_2_‐RNP (MG‐RNP) was coated with a layer of CaCO_3_, endowing MG‐RNP@CaCO_3_ with good biocompatibility and pH‐responsive degradability. In the acidic TME, the released GOx catalyzed glucose to generate H_2_O_2_, which further reacted with MnCO to produce toxic CO for gas therapy. Meanwhile, the released Ca^2+^ from CaCO_3_ decomposition elevated mitochondrial Ca^2+^ level, resulting in Ca^2+^‐driven ROS formation and apoptosis pathway activation. On the other hand, the overexpressed GSH in tumor cells would cleave the disulfide bond and release RNP, which knocked down the Nrf2 gene effectively and sensitized tumor cells to achieve enhanced CO gas therapy. Although GOx was not used as the direct template for the synthesis of CaCO_3_, the introduction of the powerful CRISPR/Cas9 gene‐editing tool is a good example for combining GOx‐related cancer therapy with gene therapy, which may stimulate more researches in this field.

### 3.3 Diabetes management

Diabetes is a widely spread metabolic diseases featured with hyperglycemia, which related to some serious complications, threaten the life and even cause death.^[^ ^53^ ^]^ In 2021, there are 537 million adults (10.5%) aged 20−79 years diagnosed with diabetes worldwide and is predicted to be 783 million (12.2%) in 2045.^[^ ^54^ ^]^ Diabetes is one of the main global killer caused 6.7 million deaths in 2021—1 person dies of it every 5 s. Diabetes is divided into two types: type 1 (T1DM) and type 2 diabetes (T2DM). T1DM is featured as an autoimmune disorder and the incidence of it peaks in childhood, while T2DM is more common with adults and the incidence of it peaks in middle and old people. T2DM is an insulin‐dependent disease featured with abnormal glucose metabolism, which is influenced by many factors including age, obesity, sedentary behavior, dietary changes etc. INS administration is the primary treatment of diabetes, and glucose responsive GOx‐based carrier can mimic the dynamic biological process of insulin secretion by *β*‐cells to maintain the stability of blood glucose, is promising for personalized treatment.^[^ ^55^ ^]^ For instance, Jiang et al. reported a GOx&ISN@CaCO_3_–TA–HA capsule for controlled INS release.^[^ ^47^ ^]^ GOx and ISN were encapsulated in CaCO_3_ spheres, then the GOx&ISN@CaCO_3_ spheres were coated with tannic acid (TA) and further conjugated with polyethyleneimine (PEI) to obtain the capsules. When incubated with glucose aqueous solution, GOx could convert glucose into gluconic acid, which caused the enrichment of protons (H^+^) and decrease of pH values, resulting in the disassembly of capsules by breaking C═N bonds between TA and PEI. Finally, 73.1% of INS was continuously released within 3 h in a glucose‐responsive manner.

**FIGURE 11:**
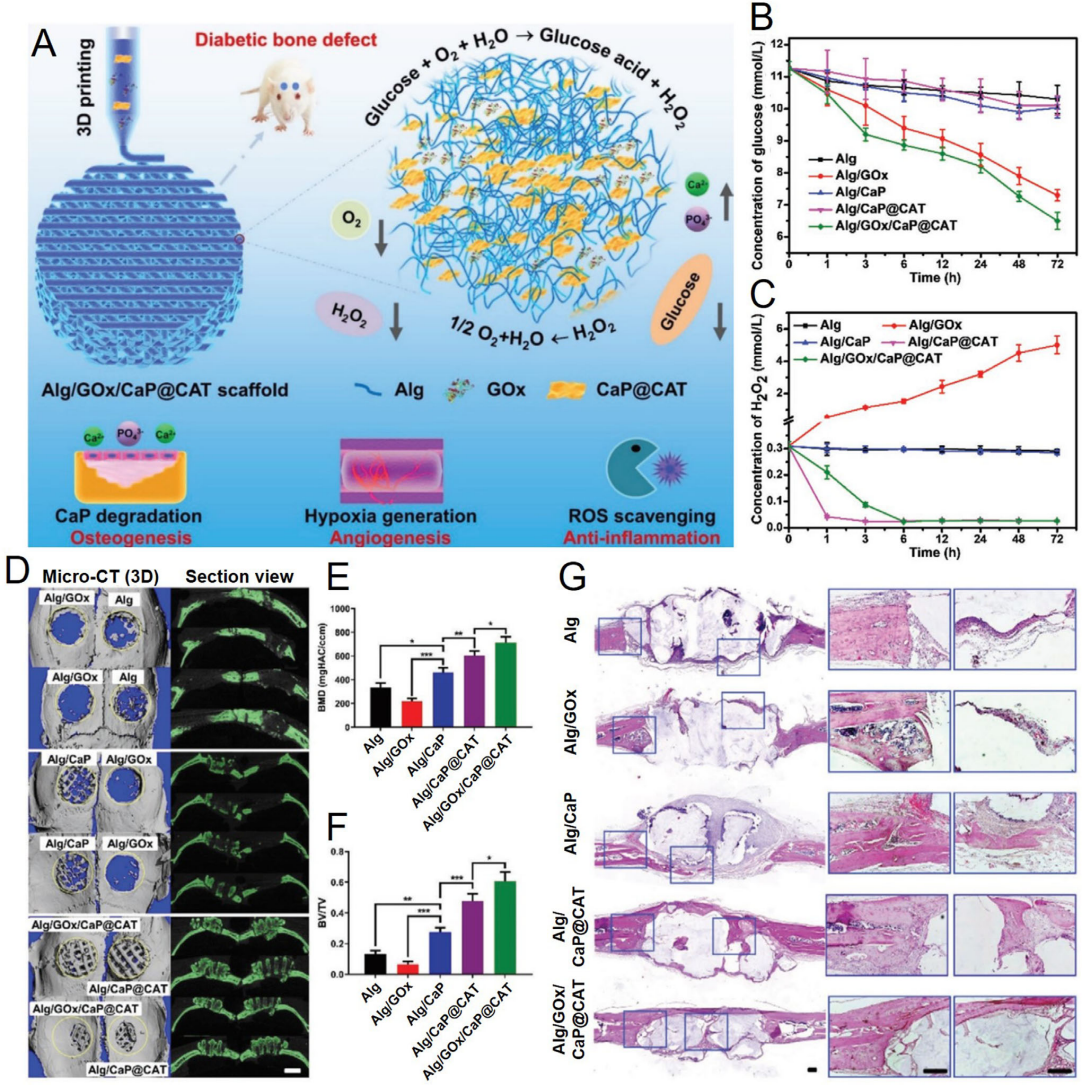
Synthesis, characterization, and application of 3D printed Alg/GOx/CaP@CAT scaffold. (A) Schematic illustration of 3D printed Alg/GOx/CaP@CAT scaffold for diabetic bone tissue regeneration. (B) The glucose and (C) H_2_O_2_ concentration variations after incubated with 3D printed scaffolds. (D) 3D X‐ray microcomputed tomography (Micro‐CT) images, section view, and the corresponding quantitative analysis of (E) BME and (F) BV/TV after implantated these scaffolds in diabetic calvarial defect for 8 weeks. (G) H&E staining of newly formed bone tissues. Reproduced with permission.^[^ ^10c^ ^]^ Copyright 2021, Wiley‐VCH.

Besides the commonly reported complications including neuropathy, coma, seizures, kidney failure, vision impairment, and cardiovascular disease, the patients with T2DM also suffer from higher risk of fracture due to impaired bone strength.^[^ ^56^ ^]^ The T2DM bone healing time is longer than that of healthy people with some determinants such as weakened osteogenesis, decreased angiogenesis and increased inflammation response in the continuous hyperglycemia environment, making it difficult to treat diabetic bone defect. Yang et al. constructed an enzyme‐functionalized scaffold (named as Alg/GOx/CaP@CAT) by 3D printing technology, this scaffold with good osteogenesis, angiogenesis, and anti‐inflammation activities were utilized for effective regeneration of diabetic bone defect (Figure 11A).^[^ ^10c^ ^]^ The CaP@CAT nanosheets (NSs) were prepared by precipitation method, then mixed with sodium alginate (Alg) and GOx for 3D printing followed by adding of CaCl_2_ solution to stimulate crosslinking. After incubated Alg/GOx/CaP@CAT scaffold in glucose solution for 72 h, the concentration of glucose decreased from 11.2 mm (diabetic level) to 6.5 mm (normal level) (Figure 11B). On the contrary, the highest H_2_O_2_ concentration (4.73 mm) was detected with Alg/GOx, and Alg/GOx/CaP@CAT maintained H_2_O_2_ concentration at a very low level (Figure 11C), these results were attributed to the cascade reactions between GOx and CAT (Figure 11A). The in vivo osteogenesis of these scaffolds was evaluated on a rat calvarial defect model in T2DM. After 8 weeks of implantation, the Alg/GOx/CaP@CAT scaffold had significantly promoted new bone formation (Figure 11D), which was confirmed by the quantitative analysis of bone mineral density (BME) and bone volume/total volume (BV/TV) value (Figure 11E,F), suggesting these defects were reconstructed well. H&E staining further demonstrated that Alg/GOx/CaP@CAT scaffold could induce more newly formed bone tissue than other scaffolds (Figure 11G). Although CAT rather than GOx was used as template to induce biomineralization of CaP NSs here, it provides a proof‐of‐concept study for bone defect repair in diabetic conditions, and it is expected that the same effect will be achieved even switch the places of CAT and GOx in the preparation process of the scaffolds. Moreover, inspired by this successful attempt of diabetic bone defect regeneration, the GOx‐mineralized CaXs is expected to treat other diabetic‐related bone complications. MET is a kind of commercial oral hypoglycemic drug against T2DM, which is also found to have potential for the treatment of bone disorders.^[^ ^57^ ^]^ MET could activate the important 5′‐adenosine monophosphate‐activated protein kinase (AMPK) signalling pathway in bone physiology, which not only promote the mesenchymal stem cells (MSCs) differentiation into osteoblasts but also reduce the bone resorption and osteoclast formation.^[^ ^58^ ^]^ Therefore, the combination of MET with GOx‐mineralized CaXs may serve as an alternative for diabetic bone osteoporosis, relevant research is undergoing in our group.

Chronic wound healing is another serious complication of diabetes, since the high level of glucose provides nutrients for bacteria to produce biofilms, resulting in wound infection and delaying wound healing.^[^ ^59^ ^]^ Hence, the platforms that can simultaneously decrease the blood glucose and kill the bacteria is expected to improve the diabetic wound healing. Tang et al. reported a biomimetic miniralization hydrogel (PAA‐CaP@FTA‐GOx) as a controllable ROS generator and antibacterial platform for diabetic wound healing, of which GOx and Fe_3_O_4_/TiO_2_/Ag_3_PO_4_ NPs (abbreviated as FTA) were immobilized in polyacrylic acid (PAA)‐CaP hydrogel through a facile biomimetic mineralization method.^[^ ^18d^ ^]^ This hydrogel could degrade gradually to release GOx and FTA under physiological condition due to the precipitation/dissolution equilibrium of CaP, which was demonstrated by immersing the hydrogel in PBS solution containing different concentrations of phosphate. When the diabetic wound covered with this hydrogel, the local blood glucose concentration was effectively reduced by GOx and the generated H_2_O_2_ was converted to toxic •OH by TiO_2_, enhancing the antibacterial properties of Ag_3_PO_4_. The good antibacterial activity of this hydrogel was conducted against both Gram‐positive bacteria Staphylococcus aureus (*S. aureus*) and Gram‐negative bacteria *Escherichia coli (E. coli)* through in vitro bacteriostatic ring experiments, and in vivo experiments found that this hydrogel showed faster healing rate and more epithelialization of the wounds on diabetic mice. However, this in vivo experiments were conducted with only two groups, PBS and the hydrogel, and the wound was not infected with any bacteria before the experiment. It seems that the conclusions are vaguely since there are so many functional components in the hydrogel. In addition, the authors proposed that the TiO_2_ could catalyze H_2_O_2_ to •OH, it should not be ignored that Fe_3_O_4_ is also an effective Fenton's reagent.

**FIGURE 12:**
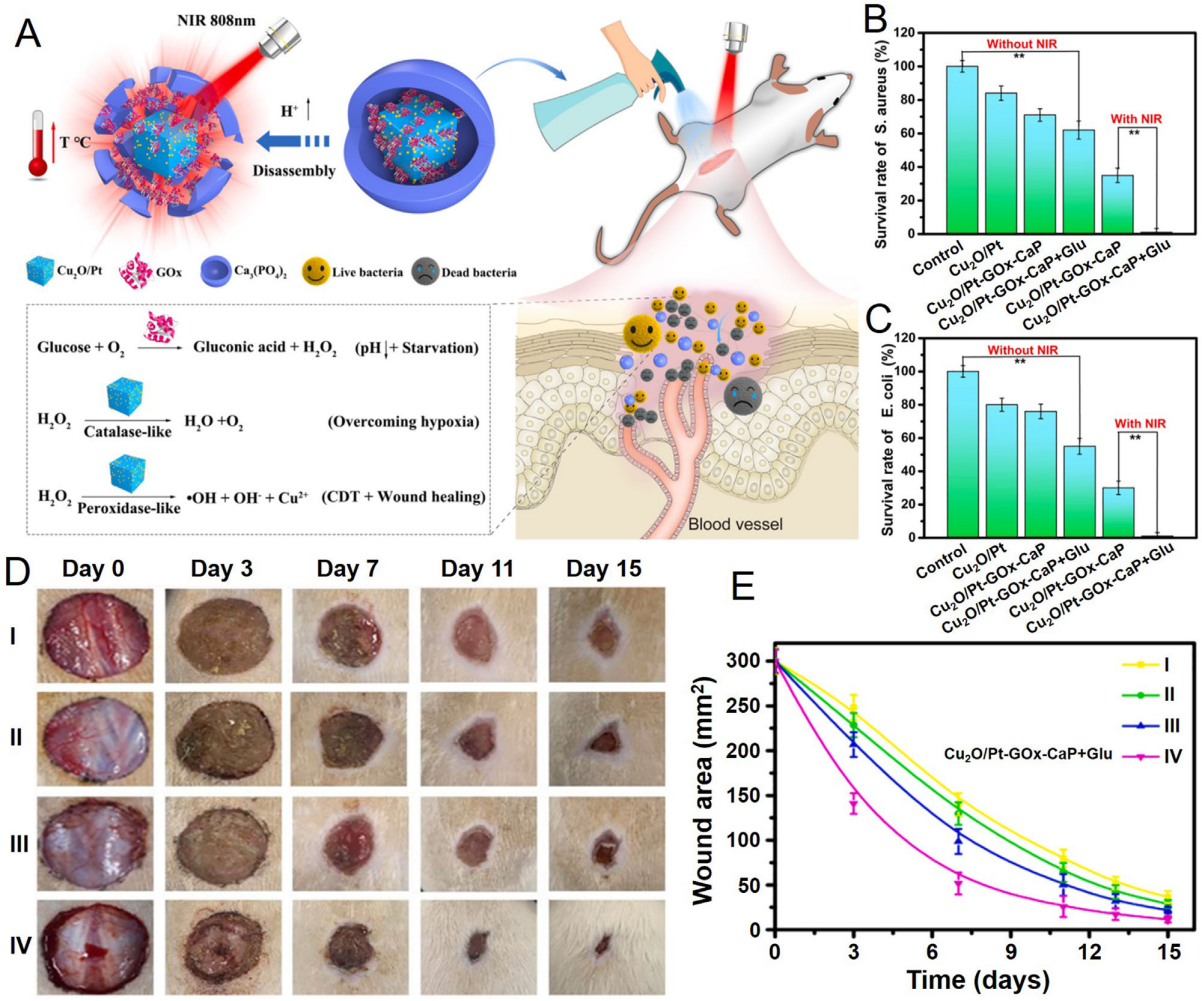
Characterization, and application of Cu_2_O/Pt‐GOx‐CaP nanoreactor. (A) Schematic illustration of Cu_2_O/Pt‐GOx‐CaP nanoreactor promoted diabetic wound healing via synergistic starvation therapy, PTT, and CDT. (B,C) The antibacterial coating plate experiments against (B) *S. aureus* and (C) *E. coli*. Glu, glucose. (D) Photographs and (E) wound area in *S. aureus* infected diabetic rats with different treatments: I, PBS; II, Cu_2_O/Pt; (III) Cu_2_O/Pt‐GOx‐CaP; (IV) Cu_2_O/Pt‐GOx‐CaP+Glu+NIR. Reproduced with permission.^[^ ^60^ ^]^ Copyright 2019, Elsevier.

In another work, Xiong et al. developed an acidity‐responsive Cu_2_O/Pt‐GOx‐CaP nanoreactor by mineralizing GOx and Cu_2_O/Pt nanozyme in DMEM to treat the *S. aureus* infected diabetic wound (Figure 12A).^[^ ^60^ ^]^ GOx triggered glucose oxidation to block the nutrients for bacteria, while the generated H_2_O_2_ could not only convert into •OH to kill the bacteria but also produce O_2_ to relieve hypoxia caused by diabetic wound infection due to the peroxidase‐ and CAT‐like activities of Cu_2_O/Pt nanozyme. The released Cu^2+^ was found to promote the proliferation and migration of NIH3T3 fibroblasts and human umbilical vein endothelial cells, which is important for wound healing. In vitro antibacterial experiments demonstrated Cu_2_O/Pt‐GOx‐CaP with a certain antibacterial ability against both *S. aureus* and E. coli (Figure 12B,C), the addition of glucose could improve the antibacterial activity. Further irradiated by an 808 nm laser, the bacteria were almost completely deleted due to the synergistic CDT/PTT/starvation therapy. In vivo diabetic wound healing assay was conducted in *S. aureus* infected type 1 diabetic rat model, and the highest healing rate was achieved when treated with Cu_2_O/Pt‐GOx‐CaP+NIR (Figure 12C,D). The reduced inflammatory cell infiltration, enhanced collagen deposition and increased compact tissue were observed in Cu_2_O/Pt‐GOx‐CaP+NIR group. In addition, the level of Ki67, anti‐VEGF and CD31 antibodies were markedly improved compared with other treatments, indicating the improved cell proliferation and angiogenesis. All of these results indicated that Cu_2_O/Pt‐GOx‐CaP+NIR could promote diabetic wound healing through antibacterial, anti‐inflammatory and pro‐angiogenic effects due to the synergetic CDT/PTT/starvation therapy, which is promising for the management of chronic wound healing.

## 4 CONCLUSIONS AND FUTURE PERSPECTIVES

GOx possess great potential for the treatment of diseases related to abnormal glucose metabolisms such as cancer and diabetes due to its specific catalytic activity against glucose. The primary issue that needs to be resolved before clinic application of GOx is effectively delivery of it to the lesions in a safe and controlled manner, since GOx often suffers from poor stability and system toxicity in vivo. CaXs with excellent biocompatibility, bioactivity and pH‐responsive biodegradability are widely used as carriers for various biomedical fields, more importantly they can be obtained by facile biomimetic mineralization process using biomolecules as template. The mineralization of GOx by CaXs is reported improve the stability, bioavailability and therapeutic efficacy of GOx and reduce its side effects due to the activatable release behavior of CaXs. Herein, we have reviewed the state‐of‐the‐art studies on GOx‐mineralized CaXs including CaP and CaCO_3_. Their synthesis, properties, and application in various biomedical applications including glucose detection, cancer treatment, and diabetes management (Tables 1 and 2) are introduced in detail. However, the application of GOx‐mineralized CaXs nanomedicine in biomedical fields is still in the preliminary stages, and there are many issues and challenges remain to be solved.

Firstly, current studies related to GOx‐mineralized CaXs are focused on CaP and CaCO_3_, other CaXs like CaSiO_3_, CaF_2_, CaS, and CaO_2_ mineralized GOx have not been reported yet. The introduction of other CaXs may provide more therapeutic chances. For instance, water‐soluble and citrate‐coated CaF_2_ nanocrystals were used for in vivo ^19^F MRI, which combined the advantages of nanocrystals (such as small size, high ^19^F equivalency, maximal ^19^F density, and surface modifiability) and ^19^F MRI tracers.^[^ ^61^ ^]^ CaS is found to gradually release Ca^2+^ and H_2_S in acidic endosomes to elevate Ca^2+^ stress in tumor cells.^[^ ^15a^ ^]^ When combined with zinc protoporphyrin (ZnPP), an inhibitor of heme oxygenase‐1, the CaS‐ZnPP effectively induced immungentic cell death through a Ca^2+^‐dependent cell death and activated antitumor immunity, which eradicated both primary 4T1 tumors and distant metastases

Secondly, the amorphous mineral like CaP is easily to incorporate foreign ions, besides the successful attempts of Mn^2+^ and Cu^2+^ doping in previous studies,^[^ ^20^ ^]^ other ions doping (e.g. Fe^2+^/Fe^3+^, Mg^2+^, Zn^2+^, Sr^2+^, etc.) with specific properties need further exploration. For instance, the classical Fenton ion Fe^2+^ can not only catalytic breakdown the endogenous H_2_O_2_ to•OH to kill cancer cells,^[^ ^62^ ^]^ but also initiate ferroptotic cell death through generation of lipoperoxides.^[^ ^63^ ^]^ Mg^2+^ level is related with various diseases including infection and cancer, which is found affected CD8^+^ T cell function via leukocyte function‐associated antigen 1 (LFA‐1), an integrin involved in T cell activation.^[^ ^64^ ^]^ Zn^2+^ is reported to enhance the cyclic guanosine monophosphate‐adenosine monophosphate synthase/interferon gene stimulator (cGAS/STING) signals, promote the infiltration of CD8^+^ T cells and improve the immunotherapy efficacy.^[^ ^65^ ^]^ Hence, it is expected that different ions doping with GOx‐mineralized CaXs may give birth to some special properties and achieve extraordinary results.

Thirdly, the immunoregulation of GOx‐mineralized CaXs nanocomplexes need to be explored. These nanocomplexes could selectively decompose in tumor tissue due to their intrinsic pH‐responsive degradability, which not only neutralize the acidic TME but also release a great amount of Ca^2+^. The elevated intracellular Ca^2+^ level was found induce immunogenic cell death, increase autophagy efficiency, and facilitate the polarization of tumor‐associated macrophages from anti‐inflammatory M2 to pro‐inflammatory M1 phenotypes.^[^ ^66^ ^]^ CaP is reported to reduce the Ado accumulation by promoting ADK‐mediated phosphorylation, inhibit Ado‐mediated immunosuppressive TME, enhance the antitumor immune response.^[^ ^30^ ^]^ The mitochondrial Ca^2+^ overload caused by CaCO_3_ and curcumin synergies even found trigger tumor cell pyroptosis, activated immune responses and suppressed tumor proliferation and lung metastasis.^[^ ^10b^ ^]^ On the other hand, lactate accumulation in TME is a major cause of immunosuppression. Exhausting glucose by GOx catalysis can not only inhibit the glycolysis of tumor cells and reduce the production of lactate, but also elevate H_2_O_2_ level and enhance the oxidative stress. Strong intracellular oxidative stress will also enhance Ca^2+^ influx through transient receptor potential channels,^[^ ^67^ ^]^ resulting in the accumulation of cytoplasmic Ca^2+^ which may promote anti‐tumor immune response of GOx‐mineralized CaXs. Hence, GOx‐mineralized CaXs may lead to a very complicated immunological effect. More recently, Lu's group found that highly activated aerobic glycolysis in human glioblastoma induces PD‐L1 expression and promote immune evasion by hexokinase2‐mediated phosphorylation of IkBa.^[^ ^68^ ^]^ Does the elimination of glucose by GOx catalysis can down‐regulation the PD‐L1 expression and enhance the immunotherapy? Although several studies demonstrated that GOx‐based nanomaterials achieved synergistic efficacy when combined with CTLA‐4, PD‐1 or PD‐L1 antibody,^[^ ^69^ ^]^ the in‐depth mechanism of anti‐tumor effect should be further studied.

Fourthly, the application of GOx‐mineralized CaXs need to be expanded. There are few works employ GOx‐mineralized CaXs to detect intracellular glucose, while the potential in detecting other biomarkers remain to be explored. In our opinion, it is not good choice of GOx‐mineralized CaXs as biosensors for glucose or other biomarkers detection, since these CaXs should decompose to ensure the contact with these biomarkers, this process may result in slow detection speed and low detection sensitivity. While it may be suitable for the long‐term monitoring of treatment process since the CaXs could maintain in the lesions for a long period of time. Considering the osteogenesis properties of Ca^2+^ and PO_4_^3−^ ions, CaP‐mineralized GOx should be a good candidate for the treatment of osteosarcoma, which may simultaneously realize the treatment of bone cancer and bone regeneration. Certainly, the bone‐targeted delivery of therapeutic nanoplatform is an essential obstacle need to overcome. To date, many moieties such as bisphosphonates, tetracyclines, bone‐targeting peptides (e.g. acidic oligopeptides containing a certain amount of aspartic acid or glutamic acid sequences, (AspSerSer)6, VTKHLNQISQSY (VTK) etc.), bone‐targeting proteins (e.g. collagen binding domains, osteocalcin, osteopontin, bone sialoprotein etc.), and bone‐targeting cells (e.g. MSCs) are found with high bone specificiy,^[^ ^70^ ^]^ these moieties can target the hydroxyapatite, collagen, osteoblasts, or the injured bone. For more details about the bone‐targeted strategies and bone‐related diseases targeting therapy, please refer to these literatures.^[^ ^70^, ^71^ ^]^ Hence, the surface modification of CaP‐mineralized GOx by the above targeting moieties may be necessary in terms of bone cancer therapy. Besides, it was reported that the biocomposite scaffolds (e.g. Fe‐CaSiO_3_) constructed by 3D printing technique exhibited sufficient mechanical support and good tumor therapeutic effect, which could repair the cortical bone defect after surgery and kill the residual tumor cells.^[^ ^72^ ^]^ These successful attempts also provide us with potential applications of CaP‐mineralized GOx biomaterials.

**TABLE 1:** Summary of GOx‐mineralized CaXs biomaterials and their application in cancer therapy.

Minerals	Reinforcement elements	Surface modification	Materials	Anti‐tumor mechanism	Cell Type	Tumor model	Mode of administration and dosage	Tumor growth inhibition	Ref.
**CaP**	*L*‐Arg	HA	GCAH	(1) GOx‐triggered glucose oxidation to starve tumor cells and produce H_2_O_2;_ (2) H_2_O_2_ oxidized *L*‐Arg to produce NO for gas therapy.	4T1, HUVEC	4T1 tumor xenograft, 50−100 mm^3^	Intravenous injections on days 1, 5, and 9; [GC], 15 mg kg^–1^ for day 1; [GC], 10 mg kg^–1^ for day 5 and 9	Completely suppressed within 14 days	^[^ ^32^ ^]^
	MET	N3‐PEG‐PO_4_, cRGD	MET@CaP‐GOx‐PEG‐cRGD	(1) GOx‐mediated glucose depletion upregulated B56δ expression, a regulatory subunit of PP2A; (2) MET downregulated the expression of CIP2A to relieve PP2A inhibition; (3) Glucose depletion coordinated with MET enhanced tumor cell apoptosis via PP2A‐GSK3β‐MCL‐1 axis.	HepG2, B16F10	HepG2 tumor xenograft, ≈80 mm^3^	Intravenously injection; 3 mg kg^–1^	Effectively inhibited, the average tumor size is ≈180 mm^3^ at day 28	^[^ ^34^ ^]^
	Cur, Obx	Alg	GOx‐Alg@CaP/Cur‐Obx	(1) GOx deprived intratumoral glucose to starve tumor cells; (2) Cur augmented mitochondrial Ca^2+^ overload to enhance therapeutic efficacy of GOx via mitochondrial dysfunction; (3) Obx inhibited cancer cell autophagy, further boosted the nutrient starvation‐dominated cancer therapy	4T1	4T1 tumor xenograft	Intravenous injection; [GOx], 2 mg kg^–1^; [Cur], 10 mg kg^–1^; [Obx], 0.25 mg kg^–1^	The growth of tumors was restrained within 14 days	^[^ ^36^ ^]^
	Mn^2+^, DOX	–	GOx‐MnCaP‐DOX	(1) Cascade reactions between GOx and Mn^2+^ ions exhausted glucose and generated abundant •OH to kill cancer cells; (2) Disturbed energy metabolism sensitived tumor cells to DOX‐induced chemotherapy.	4T1, U87MG	4T1 tumor xenograft, ≈50 mm^3^	Intratumoral injection on day 0, 3, and 7; [GOx], 3.5 mg kg^–1^; [Mn^2+^], 2.65 mg kg^–1^; [DOX], 8.68 mg kg^–1^	Effective tumor growth inhibition within 14 days	^[^ ^20a^ ^]^
	Mn^2+^, CAT, DVDMS	–	GMCD	(1) CAT catalytic decomposition of endogenous H_2_O_2_ to O_2_,which could not only replenish O_2_ for GOx catalysis but also improve production of ^1^O_2_ to enhance PDT efficacy; (2) Cascade reactions between GOx and Mn^2+^ ions exhausted glucose and generated abundant •OH to amplify oxidative stress, promote cell death.	4T1	4T1 tumor xenograft, 50−70 mm^3^	Intravenous injection; [Mn^2+^], 0.68 mg kg^–1^; [DVDMS], 2.44 mg kg^–1^	Completely inhibited or eliminated during 16 days of treatment	^[^ ^20b^ ^]^
	Mn^2+^	–	GOx@MnCaP@fibrin gel	(1) IDH1 (R132H) cells are more sensitive to the variation of glucose concentration and ROS than that of IDH1 (WT) cells; (2) Cascade reactions between GOx and Mn^2+^ ions exhausted glucose and generated highly lethal •OH to kill the ramained tumor cells.	IDH1 (R132H) U87MG	Subcutaneous U87MG tumor surgical resection model	In situ sprayed the gel at postoperative resection; [GOx@MnCaP], 2.5 mg kg^–1^	The recurrence rate decreased to 3/8 and the survival rate reached to 82.5% within 60 days	^[^ ^40^ ^]^
	Cu^2+^, DOX	PEG	PGC‐DOX	(1) The redox reaction between Cu^2+^ and GSH depleted the antioxidant GSH while Cu^2+^ reduced to Fenton reagent Cu^+^; (2) Cascade reactions between GOx and Cu^+^ ions exhausted glucose and generated highly lethal •OH to kill cancer cells. (3) Disturbed energy metabolism and redox homeostasis sensitived tumor cells to DOX‐induced chemotherapy.	4T1	4T1 tumor xenograft, ≈50 mm^3^	Intratumoral injection on day 1, 5, and 9; Intravenous injection on day 1, 4, 7, and 10; [PGC], 10 mg kg^–1^	The tumor growth inhibition rates were 94.9% and 77.8% within 14 days for intratumoral and intravenous injection, respectively	^[^ ^20c^ ^]^
**CaCO_3_**	AQDs	DSPE‐PEG	G/A@CaCO_3_‐PEG	(1) GOx catalysis blocked energy supply and downregulated the ATP‐dependent HSP expression to reverse tumor cell thermoresistance; (2) The decrease of HSP augmented the therapeutic efficacy of AQDs‐induced photothermal hyperthermia at low temperature (≈ 43°C)	SW1990	SW1990 tumor xenograft, ≈100 mm^3^	Intravenous injection on day 0, 4, and 8, and the mice were irradiated with an 808 nm laser (0.8 W cm^–^^2^, 5 min) at 12 h post‐injection; [Sb], 3.5 mg kg^–1^; [GOx], 4.5 mg kg^–1^	The tumor growth was efficiently suppressed with an inhibition rate of 83.9% within 14 days	^[^ ^48^ ^]^
	LM	PEG‐PAsp	LMGC	(1) GOx catalysis blocked energy supply and downregulated the ATP‐dependent HSP expression to reverse tumor cell thermoresistance; (2) GOx catalysis elevated the oxidative stress and acidity of TME, accelerated the decomposition of CaCO_3_ to release more Ca^2+^ ions which could disturb the ion homeostasis of mitochondria; (3) GOx‐mediated glycolysis inhibition combined with Ca^2+^‐mediated mitochondrial dyfunction enhanced tumor PTT.	CT26	CT26 tumor xenograft, ≈100 mm^3^	Intravenous injection and the mice were irradiated with an 808 nm laser (1 W cm^–^^2^, 3 min) at 4 h post‐injection	The tumor growth was effectively inhibited	^[^ ^50^ ^]^
	Fe_3_O_4_	–	GOx@CaCO_3_‐Fe_3_O_4_	(1) Cascade reactions between GOx and Fe_3_O_4_ exhausted glucose and generated •OH to kill cancer cells. (2) The introduction of ultrasound irradiation further improved tumor inhibition effect	A549	A549 tumor xenograft, ≈125 mm^3^	Intratumoral injection of the particles (200 μg mL^–1^, 100 μL); the mice were irradiated with ultrasound for 20 min (0.8 W cm^–^^2^, 1 min interval after 4 min of irradiation)	Effective inhibited the growth of tumor within 12 days	^[^ ^51^ ^]^
	MnCO, Cas9/sgRNA RNP		MG‐RNP@CaCO_3_	(1) GOx catalyzed glucose to generate H_2_O_2_, which further reacted with MnCO to produce toxic CO; (2) The released Ca^2+^ from CaCO_3_ decomposition elevated mitochondrial Ca^2+^ level, resulting in Ca^2+^‐driven ROS formation and apoptosis pathway activation; (3) The high level of GSH in tumor cells cleaved the disulfide bond and release RNP, knocking down the Nrf2 gene and sensitizing tumor cells to achieve enhanced CO gas therapy	A549	A549 tumor xenograft, ≈50 mm^3^	Intravenous injection for every 3 day for a total of 7 times, 16 mg kg^–1^	Tumor growth was greatly inhibited with 21 days	^[^ ^52^ ^]^

*Note*: CaP, calcium phosphate; GOx, glucose oxidase; *L*‐Arg, *L*‐arginine; HA, hyaluronic acid; PEG, polyethylene glycol; GCAH, GOx‐mineralized CaP loaed with *L*‐Arg and modified with HA on the surface of the NPs; MET, Metformin; PP2A, phosphatase 2A; CIP2A, cancerous inhibitor of PP2A; GSK3β, glycogen synthase kinase 3β; Cur, curcumin; Obx, obatoclax; Alg, alginates; DOX, doxorubicin; CDT, chemodynamic therapy; TME, tumor microenvironment; CAT, catalase; DVDMS, sinoporphyrin sodium; GMCD, CAT and DVDMS co‐loaded manganese (Mn)‐doped CaP mineralized GOx NPs; IDH1 (R132H), R132H mutation of isocitrate dehydrogenase 1; IDH1 (WT), IDH1 wild‐type cells; ROS, reactive oxygen species; PGC, copper (Cu)‐doped CaP mineralized PEG‐GOx NPs; GSH, glutathione; DSPE‐PEG, 1,2‐distearoyl‐sn‐glycero‐3‐phosphoethanolamine‐N‐[methoxy (poly ethylene glycol)]; G/A, GOx/2D antimonene quantum dots (AQDs); LM, liquid metal; LMGC, LM NPs absorbed GOx on its surface, then the LM‐GOx was mineralized with CaCO_3_ and further decorated with poly *L*‐aspartic acid grafted copolymer PEG‐PAsp; PTT, photothermal therapy; MG‐RNP, MnCO‐GOx‐SiO_2_‐Cas9/sgRNA ibonucleoprotein; Nrf2, erythroid 2‐related factor 2.

Finally, when in terms of clinical prospect, we believe that CaP‐mineralized GOx possesses most clinical translation potential, since the crystalline hydroxyapatite and CaP are approved by Food and Drug Administration (FDA) for use as bone graft substitutes^[^ ^73^ ^]^ and GOx with good biocompatibility and biodegradability is widely used in the food industry.^[^ ^74^ ^]^ However, there are many challenges that need to be solved before its clinical translation. To date, all of the therapeutic effect, the biosafety and long‐term toxicity of GOx‐mineralized CaXs were estimated on small animals, the mice, for a very short time. For the cancer treatment, the treatment was conducted on the xenograft tumors, the therapeutice effecacy against orthotopic and metastatic tumors remain to explore. The potential toxicity of GOx and the influence of increased intracellular ions (e.g. Ca^2+^, Cu^2+^, Mn^2+^, PO_4_^3−^ etc.) from CaXs degradation should be taken into account. In addition, appropriate surface modification may help to reduce the risk of premature released GOx, other mode of administration like microneedle patch transdermal drug delivery system may help to improve the delivery efficiency and reduce the dosage. In a word, there are still many problems to be solved, but GOx‐mineralized CaXs have opened a new avenue in biomedical fields which deserves further exploration.

**TABLE 2:** Summary of GOx‐mineralized CaXs for diabetes.

Minerals	Materials	Animal model	Therapeutic mechanism	Efficacy	Ref.
CaCO_3_	GOx‐ISN@CaCO_3_@TA‐PEI capsules	–	GOx converted glucose into gluconic acid, which caused the decrease of pH values and resulted in the disassembly of capsules to release INS.	–	^[^ ^47^ ^]^
CaP	3D printed Alg/GOx/CaP@CAT scaffolds	Type 2 diabetic rat calvarial defect model	(1) GOx catalysis could exhaust glucose and alleviate the hyperglycemia, while CAT could relieve inflammation by scavenging both the GOx catalysis generated H_2_O_2_ and overproduced H_2_O_2_; (2) The initiated hypoxic microenvironment could stimulate neovascularization; (3) The incorporation of CaP not only enhanced the mechanical property of the scaffolds, but also facilitated new bone formation.	Promoted significant new bone formation after implantation for 8 weeks	^[^ ^10c^ ^]^
CaP	PAA‐CaP@FTA‐GOx hydrogels	Diabetic mouse model with 5 mm diameter round skin wound injury	GOx could effectively reduce the local blood glucose concentration of the wound, and the generated H_2_O_2_ was catalyzed by TiO_2_ to produce toxic •OH, enhancing the antibacterial properties of Ag_3_PO_4_.	The hydrogel group showed faster healing rate and more epithelialization of the wounds within 8 days	^[^ ^18d^ ^]^
CaP	Cu_2_O/Pt‐GOx‐CaP nanoreactor	Type 1 diabetic rat trauma healing model with 20 mm full skin gouge and infected with *S. aureus*	(1) GOx triggered glucose oxidation to block the nutrients for bacteria, while the generated H_2_O_2_ not only converted into •OH for CDT, but also produced O_2_ to relieve hypoxia caused by diabetic wound infection due to the peroxidase‐ and CAT‐like activities of Cu_2_O/Pt nanozyme; (2) The strong absorption of Cu_2_O/Pt NPs could induce an effective PTT efficacy to improve the antibacterial activity under 808 nm NIR laser irradiation; (3) Cu^2+^ ions improved the expression of anti‐VEGF, promoted endothelial cell proliferation, migration, angiogenesis, extracellular matrix remodeling and wound healing.	Highest wound healing rate and good healing quality were achieved within 15 days,	^[^ ^60^ ^]^

*Note*: INS, insulin; TA, tannic acid; PEI, polyethyleneimine; Alg, sodium alginate; CAT, catalase; PAA, polyacrylic acid; FTA, Fe_3_O_4_/TiO_2_/Ag_3_PO_4_ NPs; NIR, near‐infrared; VEGF, vascular endothelial growth factor.

## CONFLICT OF INTEREST STATEMENT

The authors declare no conflicts of interest.
